# Pavlovian impatience: The anticipation of immediate rewards increases approach behaviour

**DOI:** 10.3758/s13415-024-01236-2

**Published:** 2024-10-28

**Authors:** Floor Burghoorn, Anouk Scheres, John Monterosso, Mingqian Guo, Shan Luo, Karin Roelofs, Bernd Figner

**Affiliations:** 1 Behavioural Science Institute, Radboud University, Nijmegen, The Netherlands; 2 Department of Psychology, University of Southern California, Los Angeles, CA USA; 3 Division of Endocrinology and Diabetes, Keck School of Medicine, University of Southern California, Los Angeles, CA USA; 4 Center for Endocrinology, Diabetes and Metabolism, Children’s Hospital Los Angeles, Los Angeles, CA USA; 5 Donders Institute for Brain, Cognition and Behaviour, Radboud University, Nijmegen, The Netherlands

**Keywords:** Intertemporal choice, Delay discounting, Present bias, Reinforcement learning, Pavlovian bias, Motivational bias

## Abstract

People often exhibit intertemporal impatience by choosing immediate small over delayed larger rewards, which has been implicated across maladaptive behaviours and mental health symptoms. In this preregistered study, we tested the role of an intertemporal Pavlovian bias as possible psychological mechanism driving the temptation posed by immediate rewards. Concretely, we hypothesized that the anticipation of immediate rewards (compared with preference-matched delayed rewards) enhances goal-directed approach behaviour but interferes with goal-directed inhibition. Such a mechanism could contribute to the difficulty to inhibit ourselves in the face of immediate rewards (e.g., a drug), at the cost of long-term (e.g., health) goals. A sample of 184 participants completed a newly developed reinforcement learning go/no-go task with four trial types: Go to win immediate reward; Go to win delayed reward; No-go to win immediate reward; and No-go to win delayed reward trials. Go responding was increased in trials in which an immediate reward was available compared with trials in which a preference-matched delayed reward was available. Computational models showed that on average, this behavioural pattern was best captured by a cue-response bias reflecting a stronger elicitation of go responses upon presentation of an immediate (versus delayed) reward cue. The results of this study support the role of an intertemporal Pavlovian bias as a psychological mechanism contributing to impatient intertemporal choice.

**Supplementary Information:**

The online version contains supplementary material available at 10.3758/s13415-024-01236-2.

Daily life often confronts us with choices between small rewards delivered immediately versus larger rewards delivered later. For instance, we may be tempted to go for one more drink with friends instead of going home to be well-rested for an exam the next day. Choosing immediate small over later larger rewards, known as intertemporal impatience, has been found to be implicated across maladaptive behaviours, such as unhealthy food choice (Amlung et al., 2016; Appelhans et al., 2019; Barlow et al., 2016) and poor financial decision-making (Chabris et al., 2008; Meier & Sprenger, 2010). Moreover, an increasing body of literature points towards a critical role of impatient intertemporal choice across various mental health disorders (e.g., substance use disorders, ADHD), suggesting that it forms a transdiagnostic construct that may contribute to the development and persistence of mental health problems (Amlung et al., 2019; Lempert et al., 2018; Levin et al., 2018; Levitt et al., 2022).

Given the relevance of impatient intertemporal decisions across maladaptive behaviours and mental health problems, it is important to study the cognitive mechanisms that contribute to impatient decisions. Such knowledge provides insight into the processes that give rise to intertemporal impatience and holds the promise of providing starting points for interventions that promote long-term oriented behaviours by targeting their underlying mechanisms (Scholten et al., 2019). Little is known, however, about the cognitive mechanisms through which immediate rewards exert their temptation. Insight into these mechanisms may explain why we sometimes choose immediate small over delayed larger rewards even when the delayed reward is considered as equally or even more attractive (termed *impulsive preference reversals;* Figner et al., 2010; Grether & Plott, 1979; Lichtenstein & Slovic, 1971), or why choices between sooner-smaller and later-larger rewards elicit disproportionally more impatience when the sooner-smaller reward is available immediately, compared with when both rewards are available in the future (i.e., the *present-bias*; Benhabib et al., 2010). Existing theories have attributed immediacy temptation to a motivational (e.g., “hot” or affective) system that gives rise to impatient behaviour and that competes with a control (e.g., “cool” or deliberative) system that is required to overcome these impatient tendencies (Loewenstein & O’Donoghue, 2004; Metcalfe & Mischel, 1999). The exclusive attribution of motivational processes to one system and control processes to the other has been argued, however, to cause a *motivational homunculus problem.* That is, it fails to explain what the motivation (i.e., expected outcome) is for deploying control processes, because such motivational processes are not part of the control system. To prevent such problems, motivation and control processes must be integrated (Gladwin et al., 2011; Gladwin & Figner, 2014; see Hazy et al., 2006 for a similar discussion in working memory research).

## Pavlovian biases

The field of reinforcement learning offers a theory of behavioural control that integrates motivation and control and that provides a possible explanation of immediacy temptation. Central to this theory is the distinction between instrumental and Pavlovian control of behaviour. Instrumental control of behaviour refers to goal-directed actions to obtain rewards and/or avoid punishments, established through repeated cue-action-outcome pairing. For instance, we might learn that we should decline that drink to be well-rested tomorrow. Pavlovian control, in contrast, refers to a more rigid set of approach responses in anticipation of rewards, and withdrawal responses in anticipation of punishments, elicited by environmental cues signalling these rewards. After repeated cue-outcome pairing, anticipation of the outcome (e.g., the taste of the drink) as signalled by a cue (the sight of the drink) becomes sufficient to elicit a Pavlovian response (taking a sip).

Pavlovian and instrumental control can compete for behavioural output, and the influence of Pavlovian control on instrumental actions has been termed a *Pavlovian bias.* Robust support for the existence of such biases has been acquired using go/no-go learning tasks that orthogonalize the required instrumental action (go/no-go) and the outcome that is available upon making a correct response (winning a reward/avoiding a punishment). This orthogonalization results in four instrumental conditions or trial types that are each signalled by a unique cue: Go to win reward trials; Go to avoid punishment trials; No-go to win reward trials; and No-go to avoid punishment trials. Studies adopting this paradigm have shown that the valence of the anticipated outcome biases instrumental actions in a manner that reflects the Pavlovian response tendencies to approach reward-predictive cues and to withdraw from punishment-predictive cues. That is, the anticipation of rewards increases instrumental approach and interferes with instrumental inhibition, resulting in higher accuracy on go trials but lower accuracy on no-go trials, whereas the anticipation of punishments has the opposite effect by facilitating instrumental inhibition and interfering with instrumental approach (Algermissen et al., 2022; Algermissen & den Ouden, 2023; Cavanagh et al., 2013; Guitart-Masip et al., 2011, 2012a, b; Scholz et al., 2022; Swart et al., 2017, 2018; van Nuland et al., 2020). Thus, sometimes, Pavlovian responses interfere with instrumentally optimal behaviour, conflicting with our goals.

## Pavlovian biases in intertemporal choice

Dayan et al. (2006) were the first to suggest that a similar Pavlovian bias may lie at the heart of impatient intertemporal choice. They theorized that when confronted with a reward-predicting cue (e.g., the sight of a drink), the anticipation of this reward elicits a Pavlovian approach response that can interfere with the inhibition that is required to obtain long-term (e.g., health) goals. We go one step further by proposing that the anticipation of an immediate reward triggers a Pavlovian approach response that is stronger than that triggered by a delayed reward, even when the two rewards are matched based on the degree to which one discounts delayed rewards. More specifically, we hypothesize that the anticipation of immediate rewards increases instrumental approach but interferes with instrumental inhibition more strongly than the anticipation of delayed rewards. This could, for instance, contribute to a failure to inhibit ourselves in the face of immediate temptations, at the cost of long-term goals. The goal of the current study is to provide an empirical test of this intertemporal Pavlovian bias hypothesis, using an intertemporal version of the orthogonalized go/no-go task. By focusing on the role of reward *timing* (comparing immediate versus delayed rewards) in biasing goal-directed behaviour, our research extends on previous Pavlovian bias research, which, to the best of our knowledge, has only investigated the role of anticipated outcome *valence,* comparing rewards versus punishments (except Burghoorn et al., 2024, which will be discussed below).

By distinguishing between two types of control, the Pavlovian impatience account bears resemblance to the dual-system theories discussed earlier. However, in contrast to these theories, it does not exclusively attribute motivation or control to either system; instead, both Pavlovian and instrumental control revolve around expected outcomes, thereby integrating motivation and control and circumventing a homunculus problem. The difference between the two types of control is that whereas instrumental control learns about expected outcomes based on cues and actions (cue-action-outcome contingencies), Pavlovian control learns about expected outcomes based on cues only (cue-outcome contingencies). Therefore, Pavlovian actions are less flexible compared with instrumental actions (Dayan et al., 2006). Moreover, Pavlovian actions have been defined as inflexible, reflexive responses evoked by valence that persist even when ultimately resulting in suboptimal consequences (Huys et al., 2012). In line with this notion, we propose that by reflexively and inflexibly eliciting a stronger Pavlovian approach response, cues signalling immediate (versus delayed) rewards may give rise to impatient behaviour that is suboptimal for long-term goals.^1^

Initial evidence supporting the idea that immediate rewards enhance approach behaviour compared with delayed rewards was provided by Luo et al. (2009). They used a choice titration procedure to create participant-specific *preference-matched* immediate small and delayed larger rewards (i.e., rewards matched based on the degree of delay discounting shown in the titration) and subsequently used these rewards in a Monetary Incentive Delay (MID) task. In each trial of the MID task, participants were presented with a cue indicating the available reward on that trial, after which there was a 50% chance that a target would appear. If a target appeared, participants were required to press a button as quickly as possible. Responses were found to be significantly faster in trials in which an immediate reward was available compared with trials in which a preference-matched delayed reward was available. Moreover, increased neural activity was observed in a network that had previously been shown to be implicated in incentive value during the MID task (i.e., the superior portion of the anterior insula and putamen). As discussed by the authors, one interpretation of these findings is that they reflect a conditioned response, with the anticipation of immediate rewards increasing response invigoration.^2^ However, the study did not include no-go trials that required participants to inhibit their responses, while it is often the failure to inhibit oneself in the face of immediate rewards that characterizes intertemporally impatient behaviour. To provide a complete test of an intertemporal Pavlovian bias, it would therefore be important to assess whether the anticipation of immediate rewards increases the *probability* of approach responses, increasing instrumental approach responses but interfering with instrumental inhibition.

We previously investigated the potential effect of Pavlovian associations on instrumental go/no-go behaviour using a Pavlovian-to-instrumental transfer (PIT) task (Burghoorn et al., 2024). In this study, we did not find the reward delay associated with Pavlovian cues to influence general instrumental go/no-go behaviour towards monetary rewards, showing no evidence for a Pavlovian biasing effect of immediacy. However, using a go/no-go learning task instead of a PIT task allows us to examine Pavlovian biases on instrumental behaviour towards *intertemporal* instead of general monetary rewards and to study existing Pavlovian response tendencies in instrumental learning, instead of testing for Pavlovian effects of cues that are irrelevant to the instrumental task (Burghoorn et al., 2024).

## The present study

The go/no-go task used in the present study orthogonalizes the required action (go/no-go) and the intertemporal outcome that is available upon giving the correct response (immediate/delayed). This results in four conditions: Go to win immediate reward; Go to win delayed reward; No-go to win immediate reward; and No-go to win delayed reward. In line with Luo et al. (2009), the immediate and delayed rewards were preference-matched per participant using a choice titration task, allowing us to test for the effects of immediacy while keeping the subjective value across the immediate and delayed reward (as inferred by revealed preferences) constant. We hypothesized the anticipation of immediate rewards (compared with preference-matched delayed rewards) to increase instrumental approach behaviour and interfere with instrumental inhibition. Consequently, we predicted an increased probability of making a go response in immediate reward trials compared with delayed reward trials. We expected to observe this effect when instrumental go responses were required (go trials) as well as when instrumental no-go responses were required (no-go trials).

## Methods

The study’s research question, hypothesis, design, sample size, and the analyses were preregistered on Open Science Framework (https://osf.io/c9yk8/). The materials, data, and analysis code are also available on OSF (https://osf.io/6uqf4/).

### Participants

An a priori simulation-based power analysis showed that a sample of 200 participants would be sufficient to obtain 85–95% power to detect an unstandardized effect of reward (immediate versus grand mean) of 0.30 (on log odds scale). This effect size was based on the effect sizes obtained across two pilot studies (total *N* = 58; see Supplementary Information S1 for details of all pilot studies). For the main study, we accordingly tested 206 participants on Prolific (https://www.prolific.com/), six of whom were rejected for failing more than one of the four attention checks in the go/no-go task. To be included in the study, participants were required to be fluent in English, live in a country that uses euros as its currency (because the study rewards were presented in euros), have normal or corrected-to-normal vision, and have normal colour vision. After collecting data from these 200 participants, we performed additional data quality checks by using the preregistered exclusion criteria, resulting in the exclusion of 16 participants, and 0.39% of go/no-go trials of the remaining participants. The final sample included 184 participants (70 females, 111 males, 2 nonbinary, 1 other; *M*_age_ = 28.70, *SD*_age_ = 8.48).

The study fell under a research line that received ethics approval from the local institutional review board prior to data collection (number: ECSW-2019–153), and the study was performed in accordance with the ethical standards of the Declaration of Helsinki. Digital informed consent was obtained from all individual participants. Participation was compensated with £4.50 (Prolific uses GBPs as currency). In addition, participants took part in a performance-contingent lottery where they could win one of the rewards they earned during the go/no-go task (up to €28; described further below).

### General procedure

**Fig. 1:**
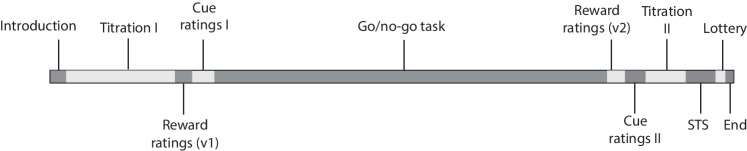
Experimental timeline. *Note.* Experimental timeline, displaying the order of tasks as administered. STS = Susceptibility to Temptation Scale. Whether the reward ratings were administered before (v1) or after (v2) the go/no-go task was counterbalanced across participants, i.e., participants only completed the task *either* before *or* after the go/no-go task. At the end of the experiment, participants were asked a few questions about their experience during the experiment (not analysed) and thanked for their participation

The experimental procedure was programmed in jsPsych (version 7.0.0; de Leeuw & Gilbert, 2023). The experiment could be completed on a desktop or laptop computer, in a Mozilla Firefox, Safari, or Microsoft Edge web browser. Figure 1 displays the experimental timeline. The complete experiment took approximately 30 min.

### Choice titration I

The titration procedure, adapted from Luo et al. (2009), served to derive a participant-specific preference-matched pair (also termed *indifference pair*) of an immediate small and delayed larger reward, later to be used in the go/no-go task. The procedure consisted of two main parts. First, participants completed the Monetary Choice Questionnaire (Kirby et al., 1999), which consists of 27 choices between an immediate reward (varying between €11 and €80, delivered on the same day) and a delayed larger reward (varying between €20 and €85 in reward amount and 7–186 days in delay), presented in a fixed order. Following the estimation procedure used by Luo et al. (2009) and Monterosso et al. (2007), choices on the MCQ were used to derive an individual discount rate using Mazur’s (1987) hyperbolic discounting model. Details of the estimation procedure are described in S2. This discount rate was used to compute the starting amount of the immediate reward in the second part of the titration procedure, which was an adaptive choice titrator. In each trial of this adaptive titrator, participants were asked to choose between an immediate reward (€X today) and a fixed delayed reward of €28 in 120 days. Possible immediate reward amounts included all even integers between €0 and €28. If a participant chose the immediate reward, the immediate reward amount on the next trial was adjusted downward with €2.^3^ If a participant chose the delayed reward, the immediate reward amount on the next trial was adjusted upward with €2. The titrator continued until a participant reached stability, reflected as a window of six trials during which the immediate reward amounts did not deviate by more than one step (i.e., €2). Participants who failed to reach stability after 50 trials were excluded from data analyses (*n* = 3).^4^ The final immediate reward amount of the participant-specific preference-matched reward pair was computed as the arithmetic mean of the immediate reward amounts on the last six trials, rounded to the nearest integer if necessary.

If, during the adaptive titrator, a participant preferred a reward of €0 today over €28 in 120 days, this trial was repeated to confirm that the participant indeed preferred this reward (termed a *confirmation trial*). If they again chose €0, the titrator ended. These participants (*n* = 1) were excluded from the data analyses, as their choices suggest that they preferred not to receive any reward, which undermines an important premise of the study. A confirmation trial was also presented if a participant preferred €28 in 120 days over €28 today. If the participant confirmed their choice, the indifference value was set at €28. These participants (*n* = 1) were not excluded from the data analyses.

The titration procedure was incentivized by informing participants that at the end of the experiment, there was a lottery where they had the chance of winning one of the rewards (see S3 for lottery details).

### Go/No-Go task

**Fig. 2:**
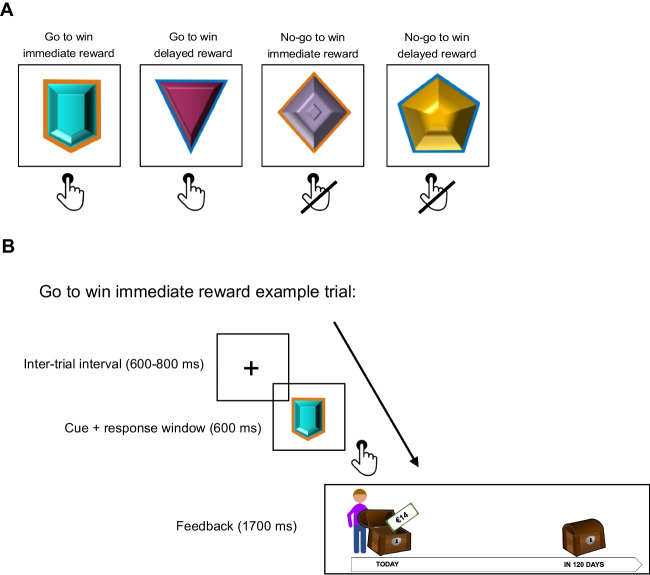
Go/no-go task. *Note.* Design of the go/no-go task. **A.** The go/no-go task consisted of four conditions, each signalled by a unique visual cue: Go to win immediate reward, Go to win delayed reward, No-go to win immediate reward, No-go to win delayed reward. The cue signalled the required instrumental action (go/no-go) and the reward available upon giving a correct response (immediate/delayed). Participants had to learn the required instrumental action by trial and error; the reward was instructed through the coloured edge around the cue, and could also be learned by trial and error. Cues were randomly assigned to conditions, and which cue edge (blue/orange) indicated which reward (immediate/delayed) was counterbalanced across participants. Each condition was presented 50 times in pseudorandom order (the same condition could not be presented more than twice in a row). **B.** Example of a Go to win immediate reward trial. The trial started with a fixation cross (inter-trial interval), after which the cue was presented. Upon cue presentation, participants had to respond within 600 ms, after which feedback was provided. If participants made a correct response for an immediate reward cue (such as that presented in **B**), the immediate reward was presented to go into a chest close to the participants’ agent on a timeline. If participants made a correct response for a delayed reward cue (not presented in **B**), the delayed reward was presented to go into a chest far away from the participants on the timeline. If participants made an incorrect response (regardless of the cue), both chests remained closed. Feedback was probabilistic; in only 80% of the trials, the feedback presented corresponded to the correctness of participants’ response

Figure 2 displays the design of the orthogonalized go/no-go task. The task, adapted from Scholz et al. (2022) and inspired by Guitart-Masip et al. (2011), was framed in terms of a gem game. Each trial of the task started with a fixation cross (600–800 ms, jittered), followed by the presentation of one of four gems (i.e., 4 cues). Participants had to learn by trial and error which gems to collect (go response) and which gems to leave behind (no-go response). For two of the gems, a correct response was rewarded with the delayed larger reward of €28 in 120 days, and for the other two gems, a correct response resulted in the participant-specific preference-matched immediate small reward. The orthogonalization of the required response (go/no-go) and the available reward (immediate/delayed) resulted in four conditions, each signalled by a unique gem (i.e., cue): Go to win immediate reward trials; Go to win delayed reward trials; No-go to win immediate reward trials; and No-go to win delayed reward trials. The required response and available reward for each cue could be learned by trial and error. However, to increase the salience of the reward (immediate/delayed) and to ensure that any reward effects could be observed from the first trial onwards, the available reward was also instructed through the coloured edge around the cue (following Scholz et al., 2022; Swart et al., 2017; van Nuland et al., 2020). Prior to the task, participants were instructed which edge colour (orange/blue) signalled which reward (immediate/delayed).

On each trial, the cue was presented for 600 ms. During this window, participants could either make a go response (by pressing the space bar) or a no-go response (by doing nothing). Participants were instructed to respond as quickly as possible for go cues. After the 600 ms response window, feedback was provided for 1700 ms. The feedback was presented by showing an agent, representing the participant, standing on a timeline with two reward chests. One of the reward chests was close to the participant on the timeline; the other chest was 120 days away. If the participant won an immediate reward, this was shown to go into the chest close to the participant. If the participant won a delayed reward, this went into a chest that stood 120 days away. If the participant made an incorrect response, both chests remained closed. Outcomes were probabilistic; on each trial, there was a 20% probability that the feedback presented to the participant did not correspond to the correctness of the response. Nevertheless, all actual responses were stored and used for the lottery at the end of the experiment. Each of the four conditions was presented 50 times in pseudorandom order, with the constraint that the same condition could not be presented more than twice in a row. Cues were randomly assigned to conditions, and which edge (orange/blue) signalled which reward (immediate/delayed) was counterbalanced across participants. The 200 trials were divided in four blocks of 50 trials, divided by 20-s breaks.

After the task instructions and before the start of the task, participants completed five practice trials in one randomly determined condition. This five-trial practice loop was repeated until participants reached 80% performance. Participants who needed more than six practice loops to complete the practice phase were excluded from the data analyses (*n* = 1).

After every 25 trials of the task, participants were presented with one of the cues and were asked to choose which of the two rewards (immediate/delayed) they would receive upon giving a correct response for that cue. The purpose of these query trials was to keep participants active and to remind them about the reward signalled by the cue. The task included eight query trials, with each cue being presented twice in random order. The task additionally included four attention checks. In these trials, a target was presented on the screen for 1000–2000 ms (jittered), and participants were instructed to press the response key upon disappearance of the target within 1500 ms. Participants were instructed about these attention checks before the task. The occurrence of the attention checks was pseudorandomly determined, with a minimum of 25 go/no-go trials between each attention check.

To incentivize task performance, participants were informed that there would be a lottery at the end of the experiment, where they had the chance of winning the outcome they received on one randomly selected go/no-go trial (hereby incentivizing response accuracy) and that their response speed increased their chance of winning the lottery (hereby incentivizing response speed). See S3 for a detailed description of the lottery.

### Secondary measures

The titration procedure and the go/no-go task described above formed the primary tasks of the experiment. In addition, we administered several other short tasks, described next.

#### Reward ratings

The go/no-go task included an immediate and delayed reward that were matched on revealed preferences using a choice titration task. To examine whether these two rewards were also valued similarly when evaluated individually (as opposed to in a choice context), we asked participants to rate how attractive they found each of the two rewards on their own. Ratings were provided using a slider on a visual analogue scale ranging from very unattractive (0, left endpoint) to very attractive (100, right endpoint). Following Figner et al. (2010), the left endpoint additionally included as anchor a delayed reward that was €1 lower in amount than the immediate reward of the indifference pair, and 1 day longer in delay than the delayed reward of the indifference pair (121 days), hereby representing a relatively very unattractive reward. The right endpoint included as anchor an immediate reward that was €1 higher in amount than the delayed reward of the indifference pair (€29), representing a relatively very attractive reward. The two rewards were presented in random order. Whether the reward rating task was completed before or after the go/no-go task was counterbalanced across participants.

#### Cue ratings

To examine preexisting differences in subjective valuation of the cues used in the go/no-go task, we asked participants to rate how attractive they found each of the cues. Ratings were provided on a visual analogue scale ranging from very unattractive (0, left endpoint) to very attractive (100, right endpoint). The cues were presented in random order. To explore whether the go/no-go task influenced the cue ratings, we again asked participants to rate the cues after completion of the go/no-go task. The cues were again presented in random order.

#### Choice titration II

To examine whether the degree of intertemporal impatience, as assessed by using the first choice titrator, remained stable across the experiment, we also administered a shortened version of the choice titrator after the go/no-go task. This version of the task only included the adaptive choice titrator. The starting value of the immediate reward was identical to that in the choice titrator administered before the go/no-go task.

#### Susceptibility to temptation scale

We administered the Susceptibility to Temptation Scale (STS; Steel, 2010) to explore whether any intertemporal Pavlovian bias effects would be associated with self-reported susceptibility to immediate gratification in daily-life. This short questionnaire (see S4) consists of 11 items scored on a 5-point scale (0 = Not true to me, 1 = Not usually true for me, 2 = Sometimes true for me, 3 = Mostly true for me, 4 = True for me). The psychometric properties (convergent, discriminant, and factor validity, and internal consistency) of the STS have been evaluated as good (Rozental et al., 2014; Steel, 2010). We added one attention check item to the scale, stating, “This is an attention check. Please select ‘Not usually true for me.’” Participants who failed this attention check were excluded from data analyses involving the STS (*n* = 2).

### Data analyses

#### Statistical models

We analysed the data using Bayesian mixed-effects models, using the package brms (Bürkner, 2018) in R (R Core Team, 2022). In our main statistical model, responses (go/no-go) on the go/no-go task were analysed as a function of the required action (go/no-go), the available reward (immediate/delayed), task block (1–4, modelled as centered linear predictor), and their interactions as fixed effects, using a Bernoulli distribution to account for the trial-level dependent variable. We accounted for by-participant random variation using a maximal random-effects structure in all analyses, as recommended by Barr et al. (2013) and Yarkoni (2020). We included a random intercept and random slopes of all fixed effects, all varying over participants, as well as all possible random correlations. The statistical models for the secondary analyses are specified in the respective results or *Supplementary Information* sections. Categorical predictors were coded using sum-to-zero contrasts and continuous predictors were mean-centered. For all analyses, we used brms’ weakly informative default priors. Despite using a Bayesian statistics package to run our models, we reported the statistical significance of the estimated effects. Effects were denoted as statistically significant when the 95% credible interval, more specifically, the 95% highest density interval (HDI) did not include 0. Reported HDIs were rounded to two decimal places, except when an HDI boundary rounded in this way was 0.00; in this case, more decimal places are reported. The estimated marginal means and 95% HDIs reported along with the results of the statistical model that tested the difference between these means were derived using the emmeans package (Lenth, 2019). Visualizations of the results were created using the packages brms and ggplot2 (Wickham, 2016). For all figures based on raw data, the displayed 95% CIs refer to confidence intervals (CIs) instead of HDIs.

#### Reinforcement learning models

To examine the computational mechanisms that may underlie the hypothesized behavioural patterns, we fitted a series of increasingly complex reinforcement learning models. We hereby followed previous work studying Pavlovian biases for rewards versus punishments (Guitart-Masip et al., 2012b; Swart et al., 2017, 2018), examining whether similar computational mechanisms apply to intertemporal rewards. We started with a Rescorla-Wagner model as base model, M0:

1 $${w}_{t}\left({a}_{t},{s}_{t}\right)={Q}_{t}\left({a}_{t},{s}_{t}\right)={Q}_{t-1}\left({a}_{t},{s}_{t}\right)+\alpha \left({r}_{t-1}-{Q}_{t-1}\left({a}_{t},{s}_{t}\right)\right)$$

In this model, action weights (*w*_*t*_) are fully determined by action values (*Q*_t_). Action values are updated on a trial-by-trial basis, based on prediction errors: the discrepancy between the expected (*Q*_*t-1*_) reward and the obtained reward (*r*_*t*-1_), scaled by the learning rate α. Action weights were transformed into go response probabilities (*p*) using a softmax function. They are scaled by the inverse temperature parameter τ, capturing response stochasticity, i.e., the degree to which responses were determined by the action weights:

2 $$p\left({a}_{t},{s}_{t}\right)=\left[\frac{\text{exp}\left(\uptau {w}_{t}\left({a}_{t}|{s}_{t}\right)\right.}{{\sum }_{a^{\prime}}\text{exp}\left(\uptau {w}_{t}\left(a^{\prime}|{s}_{t}\right)\right.}\right]$$

In M1, the softmax function was expanded by adding a parameter ξ that captures irreducible noise in action selection, due to, e.g., attentional lapses:

3 $$p\left({a}_{t},{s}_{t}\right)=\left[\frac{\text{exp}\left(\mathrm{\uptau} {w}_{t}\left({a}_{t}|{s}_{t}\right)\right.}{{\sum }_{a^{\prime}}\text{exp}\left(\mathrm{\uptau} {w}_{t}\left(a^{\prime}|{s}_{t}\right)\right.}\right]\left(1-\upxi \right)+\frac{\upxi }{2}$$

Next, in M2, we added a go bias parameter *b* to the computation of the action weight, capturing people’s general tendency to give go responses:

4 $$w_t\left(a_t,s_t\right)=\left\{\begin{array}{cl}Q_t(a_t,s_t)+b&if\;a_t=go\\Q_t(a_t,s_t)&else\end{array}\right.$$

M3 captures the hypothesized Pavlovian bias by means of a cue-response bias parameter π, which increases the weight of go responses upon presentation of a cue signalling an immediate reward, and decreases the weight of go responses in the presence of a cue signalling a delayed reward:

5 $$w_t\left(a_t,s_t\right)=\left\{\begin{array}{cl}Q_t(a_t,s_t)+b+\;\mathrm{\pi} V(s_t)&if\;a_t=go\\Q_t(a_t,s_t)&else\end{array}\right.$$

*V*(${s}_{t}$) represents the reward signalled by the cue. Because this reward was instructed by the coloured edge around the cue, we expected any effects to appear from the first trial onwards. Therefore, we fixed the values of *V*(${s}_{t}$) at 1 for immediate rewards, and −1 for delayed rewards, following previous Pavlovian bias work that used static *V*(*s*_*t*_) values to represent instructed outcome identities^5^ (Scholz et al., 2022; Swart et al., 2017, 2018; van Nuland et al., 2020). M3 assumes that the hypothesized increase in go responses in immediate reward trials is driven by a cue-response bias, with cues signalling the prospect of immediate rewards eliciting go responses. An alternative computational mechanism that could underlie the increase in go responses revolves around a learning bias. This reflects the possibility that people find it easier to learn to make a go action if that action is followed by an immediate reward than a delayed reward, whereas the opposite is the case for no-go actions. The enhanced learning if a go response is followed by an immediate reward, and if a no-go response is followed by a delayed reward, is reflected in M4 by an increased learning rate α_0_:

6 $$\mathrm{\alpha}=\left\{\begin{array}{ll}\mathrm{\alpha}_0&if\;(a_t=go\;\&\;r_t=immediate)\;or\;(\mathrm{\alpha}_t=nogo\;\&\;r_t=delayed)\\\mathrm{\alpha}_1&\;\;\;\;\;\;\;\;\;\;\;\;\;\;\;\;else\end{array}\right.$$

Finally, in M5, we included both the cue-response bias and the learning bias, hereby combining M3 and M4.

##### Model fitting, comparison, and validation

The models specified above were fitted using maximum a posteriori (MAP) estimation, which aims to find the participant-specific posterior mode. The learning rate and irreducible noise parameters were constrained between 0 and 1, the inverse temperature was constrained between 0 and 50, and the go bias and Pavlovian bias parameters were constrained between −3 and 3. A *Gamma*(3,0.3) prior was used for the inverse temperature parameter, and a *Gaussian*(0,1) prior was used for the go bias and the cue-response bias parameters. Parameters were optimized with a differential evolution algorithm implemented in the DEoptim package (Mullen et al., 2011). Models were compared by using Aikaike’s Information Criterion (AIC), with smaller values indicating a better fit, and model frequency, the proportion of participants for which each model had the lowest AIC. As recommended by Wilson and Collins (2019), we validated the best-fitting models using parameter recovery, model recovery, and posterior predictive checks.

## Results

### Go/No-Go task

**Fig. 3:**
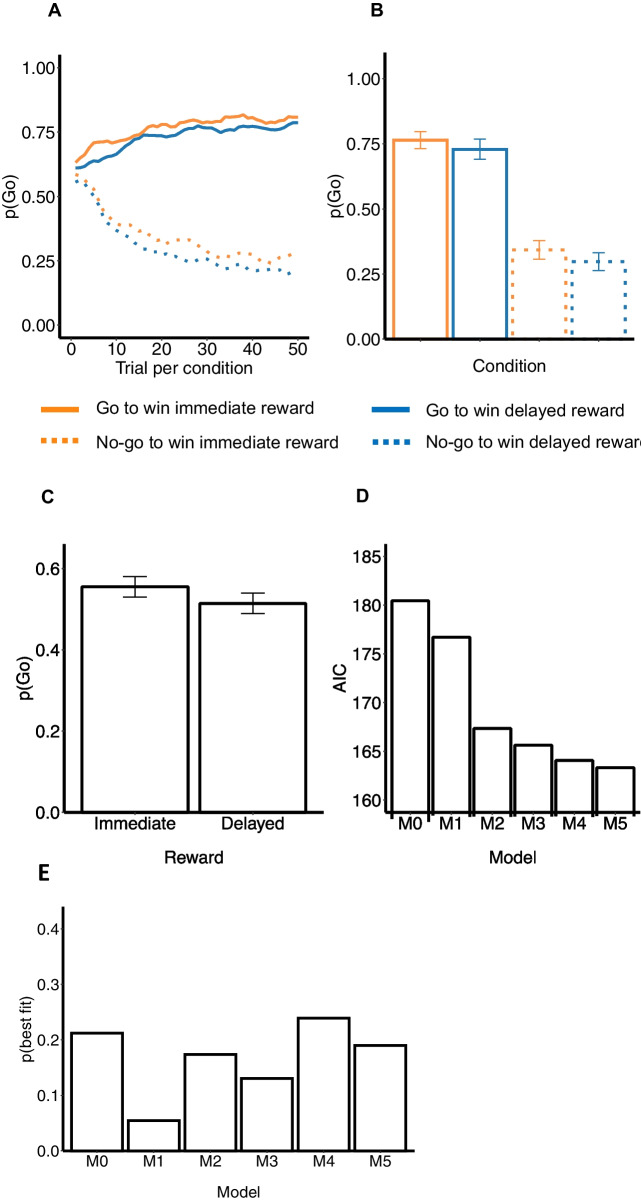
Results go/no-go task. *Note.* Results of the go/no-go task. **A.** Average trial-by-trial probability of making a go response (with 95% confidence intervals [CIs]), per condition. **B.** Average proportion of aggregated go responses (with 95% CIs) per condition. **C.** Average proportion of aggregated go responses (with 95% CIs) per available reward, aggregated over go and no-go trials. Panels **A-C** are based on raw data, which may deviate from the model-based estimated marginal means reported in the text. The reason for this is that the model uses a logit link to account for the non-linear association between the predictors and the raw binary responses (go/no-go), and because we back-transformed the resulting model-based means from the log-odds scale to the probability scale to facilitate interpretation. **D.** Model fit of the five reinforcement learning models, using the median Aikaike’s Information Criterion (AIC) across participants as measure of model fit. Lower values indicate better model fit. **E.** Model frequency, displayed as the proportion of participants for which each model had the lowest AIC value

Figure 3A displays the trial-by-trial probability of making a go response per condition. Figures 3B-C display the aggregated probability of making a go response per condition (Fig. 3B) and per reward (Fig. 3C).

#### General task performance

On average, participants showed accurate task performance, as they made significantly more go responses in go-trials than in no-go trials (go trials: *M*_pGo_ = 0.85, 95% HDI [0.82, 0.88]; no-go trials: *M*_pGo_ = 0.22, 95% HDI [0.18, 0.26]; *b*_GovsGrandMean_ = 1.52, 95% HDI [1.33, 1.72]; Fig. 3A). There was a statistically significant interaction between the required action and the task block (*b*_GovsGrandmean*Block_ = 0.60, 95% HDI [0.50, 0.69]), such that, across blocks, participants made increasingly more go responses in go-trials (*b*_Block_ = 0.53, 95% HDI [0.40, 0.66]) and fewer go responses in no-go trials (*b*_Block_ =  −0.67, 95% HDI [−0.77, −0.56]). This reflects an improvement in accuracy over the course of the task, for both go and no-go trials. As described in detail in S5, individual differences in accuracy did not moderate the Pavlovian bias effect.

#### Pavlovian bias effect

In line with the intertemporal Pavlovian bias hypothesis, participants made more go responses in immediate reward trials than in delayed reward trials, reflected by a statistically significant effect of reward (immediate: *M*_pGo_ = 0.60, 95% HDI [0.55, 0.65]; delayed: *M*_pGo_ = 0.52, 95% HDI [0.46, 0.57]; *b*_ImmvsGrandMean_ = 0.17, 95% HDI [0.03, 0.31]). A nonsignificant interaction between the reward and the required action (*b*_ImmvsGrandMean*GovsGrandMean_ =  −0.04, 95% HDI [−0.12, 0.04]) indicated that the reward effect was not significantly different in go versus no-go trials. Nevertheless, when examining this effect in both trial types separately, the reward effect only reached statistical significance in no-go trials (no-go trials: *b* = 0.22, 95% HDI [0.08, 0.36], go-trials: *b* = 0.13, 95% HDI [−0.07, 0.30]). The effect of reward did not vary as a function of task block (*b*_ImmvsGrandMean*Block_ = 0.04, 95% HDI [−0.02, 0.10]), showing no evidence that it significantly increased or decreased over the course of the task. Finally, we did not observe a statistically significant three-way interaction between the reward, required action, and task block (*b*_ImmvsGrandMean*GovsGrandMean*Block_ =  −0.04, 95% HDI [−0.09, 0.003]), indicating that the interaction between reward and required action did not vary as a function of the task block.

Next, we explored whether the reward not only increased the *probability* of making a go response, but also enhanced the *speed* with which go responses were made, taking response speed as a measure of behavioural vigour (in line with Algermissen & den Ouden, 2023; Guitart-Masip et al., 2011, 2012a; Scholz et al., 2022; Swart et al., 2017, 2018). As reported in detail in S6, however, we did not observe a statistically significant effect of reward on response times, indicating that the anticipation of immediate (versus delayed) rewards did not results in faster go responses, or vice versa. The nonsignificant reward effect also did not interact with the required action or task block.

#### Reinforcement learning models

Figures 3D-E display the model fits of the five reinforcement learning models fitted to the observed go/no-go data. Comparing the models in terms of the median AIC across participants (Fig. 3D) shows that the strongest model evidence was found for the model incorporating both a cue-response bias and a learning bias (M5). The difference in median AICs between M3 (165.62), M4 (164.07), and M5 (163.31), however, was small. We also examined the frequency with which each model was the best-fitting model per participant (Fig. 3E). Although none of the models stood out as the best-fitting model for the majority of participants, thus not resulting in a clear winner, M4 had the highest proportion of participants (23.91%) for whom it was the best-fitting model. Figure 3E also shows that for some participants, the relatively simple models M0-M2, which did not include any Pavlovian bias parameters, were the best-fitting models. These individual differences in model fit may be associated with the individual differences we observed in the Pavlovian bias effect. That is, as reported in detail in S7, whereas the simpler RL models (mostly M2) tended to be the best-fitting model more often for participants who did not show a Pavlovian bias effect, models M3-5 were the best-fitting models more often for participants who showed the hypothesized Pavlovian bias effect, and for participants who showed the opposite Pavlovian bias effect. We return to these individual differences in the discussion.

**Fig. 4:**
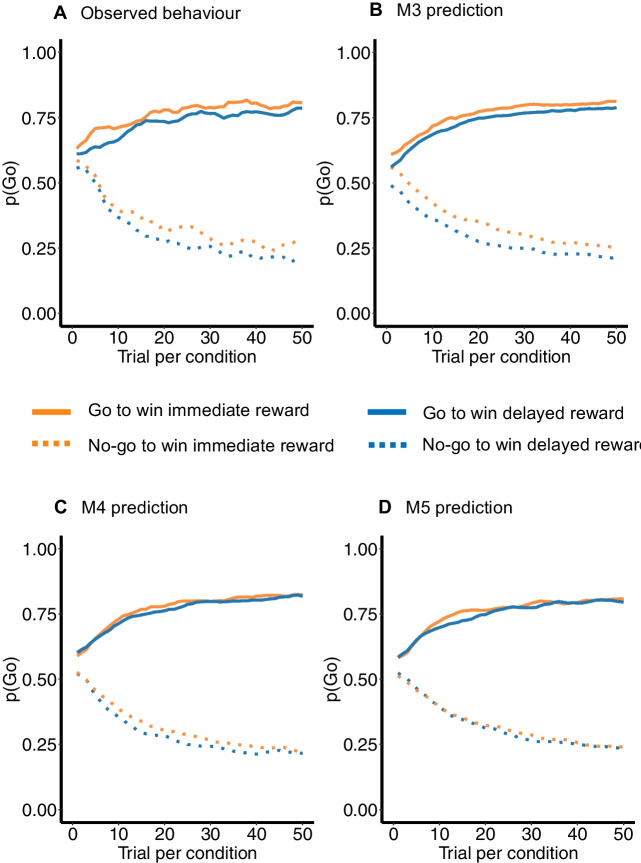
Posterior predictive checks. *Note.* Observed behaviour on the go/no-go task (**A**) and posterior predictive checks for M3 (**B**), M4 (**C**), and M5 (**D**). For each model, we simulated 1000 datasets by using the best-fitting parameters of each participant, plotted the predicted behaviour on the go/no-go task, and compared the predicted behaviour to the observed behaviour. All figures are based on raw observed (**A**) or simulated (**B-D**) data

Since our indices of model fit (AIC and model frequency) did not result in a clear winner between M3, M4, and M5, we conducted a model validation for all three models, using parameter recovery, model recovery, and posterior predictive checks (as recommended by Wilson & Collins, 2019). As reported in detail in S8, we observed consistently satisfactory parameter recovery for the irreducible noise, go bias, and cue-response bias parameters across all models, but less consistent recovery for the other parameters (e.g., the bias-congruent learning rate in M4 was not recovered^6^). Model recovery was satisfactory for M3, but less so for M4, and, in particular, M5. The observation that M5 was not well distinguishable from the other two models may not be surprising, given that M5 is a combination of M3 and M4. This indicates that despite M5’s superior model fit, as evidenced by the slight advantage in AIC (Fig. 3D), its parameter and model recovery may be compromised by its complexity. Similarly, M4’s advantage in terms of model frequency (Fig. 3E) was accompanied by a suboptimal parameter and model recovery. A third and crucial model validation criterion concerns the ability of a model to accurately generate a behavioural pattern that is similar to the observed behavioural pattern (Palminteri et al., 2017; Steingroever et al., 2014; Wilson & Collins, 2019). Therefore, we performed posterior predictive checks by simulating 1000 datasets for models M3-M5, using the per-participant best-fitted parameters. Figure 4 shows that only M3 accurately reproduced the observed data pattern. Thus, across parameter recovery, model recovery, and posterior predictive checks, we conclude that M3 exhibited the best model validation. Therefore, following the similarity in model fit between M3, M4, and M5, and the superior model validation of M3, we tentatively conclude that M3 is the winning model. A summary of the parameter estimates of M3 can be found in Table 1. A one-sample *t*-test and a one-sample Wilcoxon signed rank test showed that the Pavlovian cue-response bias parameter was statistically significantly different from zero (one-sample *t*-test: *t*(183) = 2.02, *p* = 0.045, one-sample Wilcoxon signed rank test: *V* = 10,326, *p* = 0.012). The small average magnitude of this parameter across participants is in line with the substantial interindividual variation in the effect of reward on behaviour (as discussed in detail in S7).

**Table 1:** Summary Parameter Estimates M3

Model parameter	*M*	*Mdn*	95% CI
α	0.19	0.11	[0.16, 0.22]
τ	7.33	6.91	[9.64, 7.73]
ξ	0.17	0.06	[0.14, 0.20]
*b*	0.06	0.06	[0.02, 0.11]
π	0.03	0.02	[0.001, 0.07]

Summary of the parameter estimates of the winning reinforcement learning model, M3. *M* = mean, *Mdn* = median, 95% CI = 95% confidence interval

### Choice titration I

Across participants, the immediate rewards preference-matched to a reward of €28 in 120 days ranged between €1 and €28 (raw *M* = €13.14, *SD* = €8.22). As reported in detail in S5, the intertemporal impatience shown during the titration significantly moderated the Pavlovian bias effect in the no-go trials of the go/no-go task, with stronger intertemporal impatience being associated with a stronger Pavlovian bias effect. We did not observe such a moderation in the go trials. The difference in moderation effects between go and no-go trials was statistically significant. This moderation effect did not appear to be attributable to a regression to the mean in discounting estimates (see S5 for details).

### Choice titration II

The choice titration procedure administered after the go/no-go task resulted in immediate rewards ranging between €1 and €28 (raw *M* = 13.88, *SD* = 7.99). Analysing the immediate reward amounts as a function of administration time (choice titration I / choice titration II) showed that participants became slightly, albeit significantly more patient over the course of the study (*b*_TitrationIvsGrandMean_ =  −0.37, 95% HDI [−0.58, −0.17]). A possible implication of this increased patience is that the immediate and delayed reward may not have remained preference-matched throughout the go/no-go task, with a slightly higher value of the delayed compared to the immediate reward. This, however, would have resulted in an average increase in go responding in anticipation of *delayed* (versus immediate rewards), which is the opposite of what we observed in the task. Therefore, we deem it unlikely that a drift in discounting confounded the Pavlovian bias effect reported above. We also did not observe an association between individual differences in the drift in intertemporal impatience and the Pavlovian bias effect, reflected by a nonsignificant interaction between the (centered) drift and the reward effect on go responding (*b*_ImmvsGrandMean*Drift_ =  −0.02, 95% HDI [−0.07, 0.03]). This eliminates the possibility that participants who became more impatient showed the expected Pavlovian bias effect, while participants who became more patient showed the opposite effect.

### Reward ratings

We analysed the ratings participants provided of the two preference-matched rewards as a function of reward (immediate/delayed) and administration point (before/after the go/no-go task) as fixed effects, with a random intercept for participants. Despite being preference-matched, participants, on average, rated the immediate reward significantly higher than the delayed reward (*M*_Imm_ = 73.70, 95% HDI [69.50, 77.70], *M*_Del_ = 56.60, 95% HDI [52.20, 60.60], *b*_ImmvsGrandMean_ = 8.55, 95% HDI [5.99, 11.09]). There was no statistically significant difference in ratings before and after the go/no-go task (*M*_Pre_ = 63.90, 95% HDI [59.70, 68.50], *M*_Post_ = 66.40, 95% HDI [61.70, 71.40], *b*_PostvsGrandMean_ = 1.24, 95% HDI [−1.87, 4.53]), and there was no significant interaction between the reward and administration point (*b*_ImmvsGrandMean*PostvsGrandMean_ =  −1.06, 95% HDI [−3.55, 1.47]), indicating that the effect of reward was not significantly different before versus after the go/no-go task. As reported in detail in S5, including the participant-specific difference in rating between the immediate and delayed reward in our main Pavlovian bias model showed this rating difference to moderate the Pavlovian bias effect in the go/no-go task. Post-hoc tests showed that although the direction of the Pavlovian bias effect was consistent across levels of the reward rating difference, the effect was stronger and only reached statistical significance when the immediate reward was rated higher than the delayed reward. These results point towards the possibility that the valuation differences between the immediate and delayed reward contributed to the observed Pavlovian bias effect; we return to this in the *Discussion*.

### Cue ratings

As described in detail in S9, we observed several statistically significant differences between ratings of the stimuli. By randomly assigning cues to conditions in the go/no-go tasks, we prevented these preexisting differences from confounding the Pavlovian bias effect. We observed no significant effect of administration point (before/after the go/no-go task) nor did we find any significant interactions between stimulus and administration point, indicating that the ratings in general, and the differences in ratings between stimuli, did not significantly change over the course of the experiment.

### Susceptibility To Temptation Scale

Total Susceptibility to Temptation Scale (STS) score varied between 6 and 43 (raw *M* = 22.30, *SD* = 6.89). To examine whether susceptibility to temptation moderated the observed Pavlovian bias effect of reward, we reran our main Pavlovian bias model, this time also including the STS total scores as a centered linear predictor (only as fixed effect), allowing it to interact with all other predictors. The STS scores did not interact with the main effect of reward, showing no evidence for a moderation (*b*_ImmvsGrandMean*STS_ =  −0.002, 95% HDI [−0.02, 0.02]). We also did not observe a significant main effect of STS or any other interactions involving reward and STS.

## Discussion

In the present study, we examined the effect of an intertemporal Pavlovian bias on instrumental approach/withdrawal behaviour, using a newly developed go/no-go task. In line with our hypothesis, participants were more likely to make go responses in trials in which an immediate reward was available compared with trials in which a preference-matched delayed reward was available. Thus, the anticipation of immediate rewards enhanced goal-directed approach behaviour and interfered with goal-directed inhibition. An impaired ability to inhibit ourselves in the face of immediate gratification may go at the cost of long-term goals, hereby potentially contributing to intertemporally impatient behaviour.

The Pavlovian impatience account complements currently existing descriptive intertemporal choice models by providing insight into a psychological mechanism that may drive the temptation posed by immediate rewards when controlling for the subjective value across the immediate and delayed reward (as inferred by revealed preferences). This account is strongly grounded in reinforcement learning theory, integrates motivation and control, and expands on research on Pavlovian biases in anticipation of rewards and punishments. The latter research field has found the prospect of rewards to increase approach behaviour and interfere with withdrawal, and the prospect of punishments to have the opposite effects (Algermissen et al., 2022; Algermissen & den Ouden, 2023; Cavanagh et al., 2013; Guitart-Masip et al., 2011, 2012a, b; Scholz et al., 2022; Swart et al., 2017, 2018; van Nuland et al., 2020). We show that it is not only the valence of the anticipated outcome (rewards versus punishments), but also the timing of delivery (immediate versus delayed rewards) that exerts a Pavlovian influence on instrumental approach/withdrawal behaviour.

Our results also expand on a previous study that found increased response invigoration (i.e., faster responses) in a Monetary Incentive Delay (MID) task in anticipation of immediate versus preference-matched delayed rewards (Luo et al., 2009). This study focused on response vigour on go trials, and did not test for effects on response probability (i.e., go/no-go probability) or accuracy, as the task did not include trials that required response inhibition. We show here that the anticipated reward indeed influenced response probability, as immediate rewards enhanced approach but impaired withdrawal compared with preference-matched delayed rewards. In contrast to Luo et al. (2009), however, we did not observe faster responses in the face of immediate versus delayed rewards. A possible explanation for this discrepancy is that despite being instructed to respond as quickly as possible, participants in our study may have been more concerned with accuracy compared to the study by Luo et al., which did not require participants to inhibit their response on any of the trials. The absence of an RT effect also contrasts, however, with previous Pavlovian bias studies on rewards and punishments, the majority of which reported faster responses in anticipation of rewards than in anticipation of punishments (Algermissen & den Ouden, 2023; Guitart-Masip et al., 2011, 2012a; Scholz et al., 2022; Swart et al., 2017, 2018). Almost all of these studies used longer response windows (varying between 700–1300 ms) compared with our study (600 ms), possibly allowing for more variation in response times. Algermissen & Ouden (2023), however, also used a 600-ms response window, yet reported an RT effect, suggesting that a ceiling effect in RTs is not sufficient to explain the absence of an effect (although in the study by Algermissen & den Ouden, the cues were presented 1600–2700 ms prior to the response window, giving participants more time to think about the appropriate response and therefore possibly posing less of an RT challenge). It should be noted that the reported Pavlovian bias effects of rewards versus punishments on response probability have been somewhat stronger (i.e., the median difference in go responding across nine studies was 13%) compared with the bias of immediate versus delayed rewards reported here (4% difference in go responding).^7^ This weaker Pavlovian bias effect on response probability may be accompanied by an even weaker or absent effect on response vigour.

The absence of a statistically significant interaction between the reward (immediate/delayed) and required action (go/no-go) shows that the Pavlovian bias effect was not significantly different in go versus no-go trials. In other words, anticipating immediate (versus delayed) rewards did not enhance goal-directed approach more or less strongly than it impaired goal-directed inhibition. Nevertheless, when testing for the effect of reward in go and no-go trials separately, we only observed a significant effect in no-go trials. Combined with the larger effect size in no-go trials (*b* = 0.22) than go trials (*b* = 0.13), this points towards the possibility that the intertemporal Pavlovian bias exerts a stronger effect on goal-directed inhibition than on goal-directed approach. A possibly weaker Pavlovian bias effect on go responding could result from a ceiling effect in go responses on go trials, driven by participants’ general tendency to make go responses (i.e., a go bias). Such a ceiling effect may have left little room for the go responses on go trials to be increased even further by anticipated immediate rewards, while there was ample room for go responses on no-go trials to be increased by anticipated immediate rewards. Alternative to being a methodological artefact, however, it is possible that it is predominantly goal-directed inhibition, instead of approach, that is influenced by the anticipation of immediate rewards. Indeed, many real-world instances of intertemporally impatient behaviour involve a failure to inhibit oneself in the face of an immediate reward (e.g., failing to decline a snack when offered) at the cost of long-term goals (e.g., health goals). In support of this notion, the degree of intertemporal impatience participants showed during the choice titration specifically moderated the Pavlovian bias on no-go trials, such that more impatient participants had more difficulty to inhibit their go responses in anticipation of immediate versus delayed rewards compared to more patient participants. This is consistent with the idea that a failure to inhibit oneself plays an important role in intertemporal impatience (Figner et al., 2010), as well as with the idea of intertemporal impatience as a form of impulsivity (Fenneman et al., 2022). Future research is encouraged to disentangle the role of the intertemporal Pavlovian bias in impairing goal-directed inhibition from its role in enhancing goal-directed approach.

In an earlier study, we did not find support for an intertemporal Pavlovian bias on instrumental behaviour in a Pavlovian-to-instrumental transfer (PIT) task (Burghoorn et al., 2024). In this PIT task, participants first learned to make go/no-go responses towards instrumental cues to win (non-intertemporal) monetary rewards. Next, in a separate task phase, participants learned the associations between Pavlovian cues and intertemporal monetary rewards. After this phase, participants evaluated the cues associated with larger and immediate rewards more positively than cues associated with smaller and delayed rewards, respectively, providing evidence of successful Pavlovian conditioning. In the third and final task phase, participants again performed the first (instrumental) task, but in the additional presence of the Pavlovian cues. We observed no influence of the reward delay associated with the Pavlovian cues on instrumental go/no-go responding. In the present study, however, we examined the effect of Pavlovian cues on instrumental behaviour towards *intertemporal rewards* instead of general monetary rewards. Thus, the reward delay associated with Pavlovian cues may have an outcome-specific effect on *intertemporal* goal-directed behaviour. Moreover, while in the PIT task, the Pavlovian cues were irrelevant to the instrumental task (and should therefore be ignored for optimal task performance), the cues in the present study served not only as Pavlovian cues (indicating the available reward), but also as instrumental cues (indicating the required action), and should therefore be attended for optimal instrumental performance. This allowed us to demonstrate the role of an existing intertemporal Pavlovian bias on instrumental actions.

## Computational mechanisms

To examine the computational mechanisms that may underlie the observed intertemporal Pavlovian bias on go/no-go responding, we fitted a series of increasingly complex reinforcement learning models to the data (Guitart-Masip et al., 2012b; Swart et al., 2017, 2018). Model comparison showed similar model fit for a model including a cue-response bias, with cues signalling immediate (versus delayed) rewards eliciting a conditioned go response (M3); a model including a learning bias, with enhanced learning of go responses that are followed by immediate (versus delayed) rewards (M4); and a model that combined both of these biases (M5). Model validation, however, favoured M3, showing successful parameter recovery, model recovery, and the ability to generate data that matched the observed behaviour in the go/no-go task. Therefore, we tentatively conclude that M3 is the most promising model, pointing towards a cue-response bias as the most prominent mechanism in driving the observed intertemporal Pavlovian bias. Its ability to generate data that match the observed behaviour is in line with our theory that this Pavlovian bias may contribute to intertemporally impatient behaviour. By using a model parametrization that is highly similar to that used by previous, valence-driven Pavlovian bias studies, we take a first step in showing that similar computational mechanisms apply to intertemporal rewards, and that immediate rewards elicit a stronger Pavlovian bias than preference-matched delayed rewards. At the same time, although the relatively simple models allow us to draw a clear connection with the Pavlovian bias literature, we acknowledge that we cannot say with certainty whether our Pavlovian value parameter *V*(*s*_t_) reflects a unique effect of reward delay, or whether it (additionally or alternatively) reflects a more general effect of an overall subjectively discounted reward value. We encourage future research to try to disentangle these effects by extending our models, but note that dissociating reward value from reward immediacy is conceptually complicated—we return to this issue below.

While M3 overall was the most promising candidate model, our model frequency index showed substantial individual differences in the model that formed the best fit to the data. One possible explanation for this variability is that participants may differ in the mechanisms that drive the observed Pavlovian bias. For some participants, the effect may be driven by a cue-response bias, for others it may be driven by a learning bias, and for others it may be a combination of both. An extended experimental design could be adopted to further disentangle the relative contribution of these mechanisms. For instance, Swart et al. (2017) included two types of go trials (go-left and go-right), in addition to no-go trials. If the Pavlovian bias effect is mostly driven by a conditioned go response elicited by immediacy cues (i.e., a cue-response bias), these cues should generally increase motor activation, regardless of whether a left or right response is required (i.e., without influencing accuracy on go trials). In contrast, if the Pavlovian bias effect is mostly driven by enhanced learning of go responses followed by immediate rewards, accuracy on go-left and go-right trials should increase in immediate (versus delayed) reward trials. Given that the current study was a first inquiry into intertemporal Pavlovian biases, we decided not to increase the task complexity and duration by using this extended design. However, future studies could incorporate this design to examine interindividual variability into the computational mechanisms that underlie Pavlovian biases. Finally, for some participants, even the relatively simple models without any Pavlovian bias parameters (mostly M2) were the best-fitting model. These models tended to be best-fitting models more often for participants who did not show the Pavlovian bias effect, indicating an association between individual differences in the Pavlovian bias effect and model fit.

## Strengths, limitations, and future directions

The current study has several strengths. First, the research question, hypotheses, study design, sample size, and data analyses were preregistered, and the sample size was determined a priori to achieve 85–95% power to detect the Pavlovian bias effect. Second, we extended on previous work that observed robust Pavlovian bias effects of reward *valence* (rewards versus punishments) by demonstrating the role of reward *timing*. Third, we gained insight into the computational mechanisms that may underlie the observed effect, and conducted a model validation on the three best-fitting models.

Our study has an important limitation, providing a suggestion for future research; in our design, participants who failed to inhibit their response to obtain a delayed reward did not receive any reward (neither immediate nor delayed). In daily life, however, failing to inhibit oneself in the face of temptation to achieve a long-term goal often results in an immediate smaller reward (e.g., eating that extra slice of cake despite one's original plan to reduce calorie intake to improve long-term health). We did not include this feature in our design, because it would not have allowed us to fully dissociate the anticipation of delayed rewards from the anticipation of immediate rewards (as each cue would be associated with both rewards). Having observed the Pavlovian bias effect with our current study design, however, a next step could be to increase the ecological validity of the paradigm. For instance, following O’Connor et al. (2021), one could reward unsuccessful no-go responses towards the delayed larger reward with a smaller immediate reward, hereby mimicking the situation where, e.g., a failure to stick to one’s health diet results in that extra slice of cake. Importantly, this immediate smaller reward should be below the participant-specific indifference value to ensure that this reward is less valuable than the delayed larger reward (thereby also possibly inducing a feeling of regret of having given in to their temptations, as if often the case in real-life situations). In addition to forming a conceptual replication with a more ecologically valid design, it would be interesting to examine whether, in contrast to the present study, this results in an association between the Pavlovian bias and self-reported susceptibility to temptation.

Finally, we wish to discuss several other possible directions for future research. In the current study, the immediate and delayed rewards were preference-matched per participant using an incentive-compatible choice titration procedure. This allowed us to test for the effect of reward immediacy on go/no-go responding while controlling for subjective value across the immediate and delayed reward (as inferred by revealed preferences). Nevertheless, during the reward rating task, the immediate reward was, on average, rated as more attractive than the delayed reward. Moreover, a larger rating difference was associated with a stronger Pavlovian bias effect, suggesting that these valuation differences may have contributed to the Pavlovian bias effect. This raises the question whether the observed Pavlovian bias effect reflects a conditioned response purely driven by immediacy or whether the immediate reward was considered as more valuable than the delayed reward. The latter option would be in line with a theory proposing that when the two rewards are presented in a choice context (such as the choice titration task), self-control processes increase the relative value of the delayed reward, while these self-control processes are not operative in nonchoice contexts (such as the rating task or the go/no-go task), resulting in a relatively increased value of the immediate reward (Figner et al., 2010; Luo et al., 2009).

From a methodological perspective, one could ask whether the immediate and delayed rewards used in the go/no-go task should be matched in a choice context or in a nonchoice context. Discrepancies in preferences elicited by different elicitation methods have long been recognized in the literature and are known as preference reversals (first reported by Lichtenstein & Slovic, 1971). The literature does not point to one elicitation method as most closely approximating the “true” subjective value but points towards differences between elicitation methods in considerations, weighting, valuation, and integration of inputs, and/or differences in the mapping from subjective value to observed responses (see e.g., Bettman et al., 1998; Johnson & Busemeyer, 2005; Kvam & Busemeyer, 2020; Slovic, 1995; Tversky et al., 1990; Warren et al., 2011). Any divergence in the effect of reward immediacy between elicitation methods could have important implications, as it suggests vulnerability to regret. For instance, one’s past behaviour elicited in a context in which choice was not salient could seem short-sighted when a counterfactual alternative option is made salient. An extensive discussion of this issue goes beyond the scope of this paper, but we encourage future research to include several preference-elicitation methods, enabling a more systematic investigation into the role of these methods in reward valuation and the Pavlovian bias effect. Such studies could examine whether the Pavlovian bias effects are still observed when the two rewards are matched using a nonchoice method, such as rankings, ratings, or pricings. We acknowledge that our conclusions regarding the observed effects of immediacy on go/no-go responding are limited to reward pairs that were preference-matched via a choice procedure.

From a conceptual point of view, however, we argue that even if such a study were to reveal the Pavlovian bias effect to be driven solely by reward valuation effects, the subjective value of a reward usually inherently incorporates a delay attribute (as also pointed out by Luo et al., 2009), resulting in a subjectively *discounted* reward value. Thus, the effects of immediacy and reward valuation might be two nonmutually exclusive mechanisms that are difficult to clearly dissociate. Nevertheless, future research could take an initial step by systematically varying the reward matching procedure, or by investigating the relative contributions of specific reward attributes that are assumed to contribute to the overall subjectively discounted reward value. For instance, one could include a separate Pavlovian amount variable and a Pavlovian delay variable (both of which can take on as many values as there are amount and delay levels, similar to Huys et al., 2011), allowing one to test for the effect of delay beyond the effect of reward amount and vice versa. This approach would require an extended go/no-go paradigm that orthogonalizes the reward and delay, similar to what we previously did for a Pavlovian-to-instrumental transfer (PIT) task (Burghoorn et al., 2024). By comparing the effect sizes or parameter magnitudes of the delay and amount variables, one could gain initial insight into the relative contributions of reward amount and reward delay on the Pavlovian bias effect.

Next, although intertemporal impatience can be adaptive in certain environments (Fenneman et al., 2022), it has also been proposed as a possible transdiagnostic construct that may contribute to the development and persistence of maladaptive behaviours and mental health disorders (Amlung et al., 2019; Lempert et al., 2018; Levin et al., 2018; Levitt et al., 2022). Research into an intertemporal Pavlovian bias may therefore also provide insights into the psychological processes implicated in the maladaptive behaviours and disorders characterized by intertemporal impatience. An increased Pavlovian bias driven by reward valence (i.e., rewards versus punishments) has indeed been associated with various mental health symptoms, such as mood and anxiety symptoms, suicidal thoughts and behaviours, first-episode psychosis, and substance abuse (Garbusow et al., 2022; Millner et al., 2019; Mkrtchian et al., 2017; Montagnese et al., 2020; Nord et al., 2018; Peterburs et al., 2022; but also see Albrecht et al., 2016, and Huys et al., 2016 for studies showing decreased Pavlovian biases in schizophrenia and depression, respectively). Future research is encouraged to examine the association between the strength of the intertemporal Pavlovian bias and the severity of mental health problems characterized by intertemporal impatience. If such associations are observed, an important next step would be to examine the direction of this relation, for instance by studying whether (a change in) the strength of the Pavlovian bias predicts (a change in) later mental health problems and/or vice versa.

Finally, we observed substantial individual differences in the Pavlovian bias effect, as well as in the computational model that best fitted the data. It would be of interest to understand the (neuro-)cognitive mechanisms that explain these individual differences. Research on the reward versus punishment-driven Pavlovian bias has pointed towards the role of attention regulation, with a decreased amount of attention paid to Pavlovian cues and outcomes being associated with a reduced Pavlovian bias (Algermissen & den Ouden, 2023; Garofalo & di Pellegrino, 2015; Schad et al., 2020). Others observed that increased midfrontal theta activation (Algermissen et al., 2022; Cavanagh et al., 2013; Csifcsák et al., 2020; Swart et al., 2018) and increased frontal cortical dopamine (Scholz et al., 2022) were associated with a reduced Pavlovian bias. It would be relevant to understand whether similar and/or unique mechanisms may be at play for intertemporal Pavlovian biases. Ultimately, such knowledge could provide starting points for the development of interventions to improve mental health, for instance by upregulating the mechanisms that are found to be associated with reduced Pavlovian biases on goal-directed behaviour.

## Conclusions

Using a newly developed intertemporal go/no-go learning task, we provide empirical evidence of an intertemporal Pavlovian bias on goal-directed behaviour. Anticipation of immediate rewards increased goal-directed approach behaviour and interfered with goal-directed withdrawal more strongly compared with the anticipation of preference-matched delayed rewards. Our computational models suggested that this effect may be driven by a cue-response bias, with cues signalling immediacy eliciting a Pavlovian approach response. The supported role of an intertemporal Pavlovian bias provides a mechanistic account for the temptation posed by immediate rewards that may contribute to intertemporally impatient behaviour.

## Supplementary Information

Below is the link to the electronic supplementary material.

Supplementary file1 (DOCX 2104 kb)

## Data Availability

All data and materials are available on Open Science Framework (OSF; https://osf.io/6uqf4/).

## Footnotes

1 Although the inflexibility of Pavlovian control may often result in impatient behaviour, this does not always need to be the case. As Dayan et al. (2006) point out, in some situations, long-term rewards may also elicit inflexible Pavlovian approach responses. We argue, however, that typically, immediate rewards elicit a stronger Pavlovian approach response than delayed rewards.

2 The authors’ primary explanation holds that whereas self-control processes increased the value of the delayed reward in the choice titration, these processes were not engaged in the MID task, resulting in a relatively higher value of the immediate reward. We return to the role of discrepant valuations in the discussion.

3 We deviated from Luo et al. (2009), who adjusted *k*-values upward or downward with a quarter step on a log_10_ scale. We observed that for very high or low *k*-values, this resulted in extremely small changes to *k*-values, such that the rounded immediate reward amount remained unchanged compared with the previous trial. Because we required integer reward amounts for our go/no-go task, we decided to adjust the immediate reward amount upward or downward with €2.

4 This applies only to the adaptive titrator administered *before* the go/no-go task, because the stability in this task was crucial to obtaining a preference-matched reward pair for the go/no-go task. If a participant did not reach stability in the adaptive titrator administered *after* the go/no-go task (but did reach stability in the titrator administered before the go/no-go task), they were retained in the sample (*n* = 1).

5 Fixing *V*(*s*_t_) values may raise the question how exactly these values are learned and exert their influence on actions. Because *V*(*s*_t_) was signalled by the coloured edge around the cues, its values do not need to be learned in a model-free way. Possibly, however, the Pavlovian values exert their effect in a more model-based manner, in which the outcome identities are being represented in a simplified, categorical manner (similar to the *defocused* model-based Pavlovian learning described by Dayan & Berridge, 2014). Defocused model-based Pavlovian learning therefore has been argued to be highly similar to model-free learning and may result in similar predictions. Dissociating between these types of learning is therefore complicated and beyond the scope of the current study. Because we aimed to use a computational framework that is similar to previous Pavlovian bias work, we adopted the presented computational model with fixed *V*(*s*_*t*_) values.

6 As discussed in S8, we adopted a relatively conservative approach by examining parameter and model recovery for the full range of plausible parameter values. Another common approach in the literature is to use the more limited range of parameter values determined by actually observed per-participant best-fitting parameter values. Rerunning our parameter and model recovery in this way showed improved parameter recovery for the learning rates and inverse temperature, and improved model recovery for M4 and M5. Nevertheless, the conclusion that recovery was most successful for M3 wass consistent across both approaches. We also examined whether parameter recovery for M4 would be improved with an alternative parametrization that is more similar to that used by Swart et al., (2017, 2018) and that uses a nonlinear transformation to avoid a hard boundary condition. Because this did not substantially improve parameter recovery, we retained our original model parameterisation (see S8 for details).

7 These percentages were obtained by extracting the raw means displayed in Fig. 3B and comparing these estimates to the means extracted from the same figure reported in nine previous studies (which all reported the same figure). Two additional studies, which compared the Pavlovian bias between a drug or patient group versus a control group, but did not plot the results for each group separately, were excluded. The effect sizes of these studies were 5% and 28%, respectively.

## Code availability

All code is available on OSF (https://osf.io/6uqf4/).

## Declarations

### Open practices statement

The study was preregistered on OSF (https://osf.io/c9yk8/), and the preregistration adheres to the disclosure requirements of the institutional registry. All materials, analysis code, and data are also available on OSF (https://osf.io/6uqf4/).
