# Supplementary material for: Pavlovian impatience: The anticipation of immediate rewards increases approach behaviour
Source: Cogn Affect Behav Neurosci. 2024 Oct 28;25(2):358–76. doi: 10.3758/s13415-024-01236-2 (PMC11906527; doi:10.3758/s13415-024-01236-2)
Supplement: Supplementary file 1 — Supplementary file1 (DOCX 2104 kb) [file 13415_2024_1236_MOESM1_ESM.docx]

**Supplementary Information**

**Pavlovian impatience: The anticipation of immediate rewards increases approach behaviour**

Floor Burghoorn, Anouk Scheres, John Monterosso, Mingqian Guo, Shan Luo, Karin Roelofs, Bernd Figner

**Contents**

S1. Pilot studies 2

S2. Estimation procedure discount rate 10

S3. Lottery 11

S4. Susceptibility to Temptation Scale (STS) 12

S5. Moderators of the Pavlovian bias effect 13

S6. Response times and late responses 22

S7. Individual differences in behaviour and model fit 28

S8. Parameter and model recovery 33

S9. Cue ratings 47

References 50

**S1. Pilot studies**

Before collecting data for the full study reported in the main text, we conducted four pilot studies. The goals of these pilot studies were (i) to optimise the study design based on participant feedback, data quality, and the observed effects, and (ii) to obtain an indication of the effect size for the power analysis of our full study. Since the designs of Pilot 2 and Pilot 3 were closest to that of the full study, we used the effects obtained in these two studies as input for our power analysis.

We realize it is rather unusual to report pilot studies, but we think it might be of interest to some readers, particularly researchers who might be considering conducting studies using task paradigms similar to ours. Below, we describe the main differences between the four pilot studies and the full study, and report the most important results for each pilot study. Figure S1.1 displays the results of all four pilot studies, as well as the results from the full study (for the purpose of comparison). We did not fit any computational models to the pilot study data, and therefore do not report any computational results. The pilot data and the R code used to analyse these data are available on OSF (<https://osf.io/6uqf4/>). Participants were not allowed to take part in more than one pilot study, and none of the participants of the full study took part in the any of the pilot studies.

**Pilot 1**

The three main differences between the first pilot study and the full study are that (i) the response window during the go/no-go trials was 800 ms (compared to 600 ms in the full study), (ii) two out of the four gems were different compared to the full study, and (iii) we did not administer the Susceptibility to Temptation Scale (STS; we did not administer the STS in any of the pilot studies).

Thirty-one participants took part in the first pilot study. Following our preregistered exclusion criteria, two participants were excluded from the analyses, as well as 0.38% of the go/no-go data of the remaining participants (see the R code for exclusion details). The remaining pilot sample thus included 29 participants (13 female, 15 male, 1 non-binary; *M*_age_ = 29.66, *SD*_age_ = 8.84). Figure S1.1A displays the main results of this pilot study, showing no clear influence of the anticipated reward (immediate/delayed) on go responding. Although, based on Figure S1.1A and the sample size, we did not expect to obtain a statistically significant effect of reward, we ran our main mixed-effects model to get an indication of the effect size. The model indeed showed no statistically significant effect of reward on go responding (*b*_ImmvsGreandMean_ = -0.03, 95% HDI [-0.42, 0.34]). This effect also did not interact with the required action (*b*_ImmvsGreandMean*GovsNoGo_ = -0.15, 95% HDI [-0.40, 0.10]) or the task block (*b*_ImmvsGreandMean*Block_ = -0.01, 95% HDI [-0.19, 0.18]). We also did not observe a statistically significant three-way interaction between reward, required action, and task block (*b*_ImmvsGreandMean*GovsNoGo*Block_ = *b* = -0.08, 95% HDI [-0.25, 0.07]). We did observe a statistically significant effect of required action (*b*_GovsNoGo_ = 2.46, 95% HDI [1.85, 3.05]), with more go responses in go trials than no-go trials. This effect became stronger over the course of the task, as indicated by a statistically significant interaction between required action and task block (*b*_GovsNoGo*Block_ = 0.72, 95% HDI [0.48, 0.98]). No other statistically significant main effects or interactions were observed.

**Figure S1.1**

*Results Across Pilot Studies and Full Study*

*Note.* Average trial-by-trial probabilities of making a go response (with 95% Confidence Intervals), per condition, across the four pilot studies and in the full study. All panels are based on raw data.

**Pilot 2**

In the second pilot study, we shortened the response window to 600 ms to examine whether giving participants less time to think about the appropriate goal-directed action would increase their reliance on Pavlovian responses tendencies. Moreover, compared to Pilot 1, we replaced two gem stimuli that were rated as substantially less attractive than the other two stimuli. By doing this, we aimed to create a set of stimuli that were judged as approximately equally attractive (see S8 for the differences in attractiveness ratings between these stimuli).

Similar to Pilot 1, 31 participants took part in Pilot 2. Following our preregistered exclusion criteria, two participants were excluded from the analyses, as well as 0.51% of the go/no-go data of the remaining participants. The remaining pilot sample thus included 29 participants (11 female, 17 male, 1 non-binary; *M*_age_ = 25.61, *SD*_age_ = 5.38). Figure S1.1B shows the main go/no-go patterns observed in Pilot 2, which are in support of the hypothesized effect of reward (immediate/delayed) on go responding. Running our mixed-effects model on these data showed no statistically significant effect of reward, which is not surprising given the small sample size, but the effect size was considerably larger compared to Pilot 1 (*b*_ImmvsGreandMean_ = 0.30, 95% HDI [-0.05, 0.68]). The effect of reward did not interact with the required action (*b*_ImmvsGreandMean*GovsNoGo_ = -0.15, 95% HDI [-0.14, 0.15]) or the task block (*b*_ImmvsGreandMean*Block_ = 0.01, 95% HDI [-0.25, 0.10]). We also did not observe a statistically significant three-way interaction between reward, required action, and task block (*b*_ImmvsGreandMean*GovsNoGo*Block_ = *b* = -0.04, 95% HDI [-0.13, 0.06]). Similar to Pilot 1, we did observe a statistically significant effect of required action (*b*_GovsNoGo_ = 1.70, 95% HDI [1.22, 2.19]), with more go responses in go trials than no-go trials. Also, again, this effect became stronger over the course of the task, as indicated by a statistically significant interaction between required action and task block (*b*_GovsNoGo*Block_ = 0.62, 95% HDI [0.43, 0.80]). No other statistically significant main effects or interactions were observed.

**Pilot 3**

Following the success of shortening the response window in Pilot 2, we next examined whether we could increase the effect of reward even more by further shortening the response window, without frustrating participants with the task difficulty. Therefore, we used a response window of 500 ms in Pilot 3. Moreover, after testing the first 15 participants of this pilot study, we implemented a small change that only allowed for the experiment to be run on a Safari, Mozilla Firefox, or Microsoft Edge browser, in order to avoid some technical issues that we encountered with participants using different browsers. Finally, we added a reward rating task with simplified rating anchors. Instead of using intertemporal rewards as anchors (as explained in the main text), this task used *very unattractive (0)* and *very attractive (100)* as anchors. The goal of this additional task was to compare the ratings provided in the tasks with the original and the simplified anchors. We did not observe any major differences between the two rating tasks. Moreover, when asking participants which of the two types of anchors was more sensible to them, no clear winner appeared. Therefore, we retained the reward anchors used in Pilot 1 and 2 for the full study.

Thirty-two participants took part in Pilot 3. Following our preregistered exclusion criteria, three participants were excluded from the analyses, as well as 1.07% of the go/no-go data of the remaining participants. The remaining pilot sample thus included 29 participants (8 female, 20 male, 1 non-binary; *M*_age_ = 26.34, *SD*_age_ = 6.28). Figure S1.1C displays the main go/no-go results of Pilot 3, showing a pattern similar to that observed in Pilot 2. Task performance, however, seems to be somewhat lower than in Pilot 2, possibly reflecting the increased task difficulty caused by the shorter response window. Our mixed-effects model showed no statistically significant effect of reward, with an effect size similar to Pilot 2 (*b*_ImmvsGreandMean_ = 0.31, 95% HDI [-0.04, 0.64]). The effect of reward did not interact with the required action (*b*_ImmvsGreandMean*GovsNoGo_ = 0.04, 95% HDI [-0.19, 0.28]) or the task block (*b*_ImmvsGreandMean*Block_ = -0.005, 95% HDI [-0.11, 0.11]). We also did not observe a statistically significant three-way interaction between reward, required action, and task block (*b*_ImmvsGreandMean*GovsNoGo*Block_ = *b* = -0.06, 95% HDI [-0.21, 0.11]). Again, we did observe a statistically significant effect of required action (*b*_GovsNoGo_ = 0.97, 95% HDI [0.61, 1.32]), with more go responses in go trials than no-go trials. Also, again, the effect became stronger over the course of the task, as indicated by a statistically significant interaction between required action and task block (*b*_GovsNoGo*Block_ = 0.45, 95% HDI [0.30, 0.61]). No other statistically significant main effects or interactions were observed.

Since Pilot 3 did not result in a stronger effect of reward compared to Pilot 2, we decided to use the response window from Pilot 2 (i.e., 600 ms) for the full study.

**Pilot 4**

Pilot 4 was conducted to explore an alternative strategy for increasing the effect size observed in Pilot 1. In this fourth pilot study, we aimed to increase participants’ reliance on Pavlovian response tendencies by increasing the instrumental task difficulty; not by shortening the response window (this was 800 ms in Pilot 4), but by increasing the number of gem stimuli from four to eight, with two gems per condition in the go/no-go task. Thus, we had two Go to win immediate reward gems, two Go to win delayed reward gems, two No-go to win immediate reward gems, and two No-go to win delayed reward gems. Four of the gems were identical to those used in Pilots 2 and 3, and four new gems were added.

Thirty-two participants took part in Pilot 4. Following our preregistered exclusion criteria, four participants were excluded from the analyses, as well as 0.04% of the go/no-go data of the remaining participants. The remaining pilot sample thus included 28 participants (9 female, 18 male, 1 non-binary; *M*_age_ = 37.57, *SD*_age_ = 12.85). Interestingly, the mean age of the participants was notably higher compared to the other pilot studies and the full study (median age = 40.50), for which we do not have any apparent explanation. Figure S1.1D displays the main go/no-go results of Pilot 4. Although this also shows the expected pattern of responding, with increased go responding in anticipation of immediate compared to delayed rewards, this pattern seems to be somewhat weaker compared to Pilot 2 and Pilot 3. Moreover, compared to Pilot 2 and Pilot 3, participants seemed to have taken longer to learn the task contingencies, with an increase in task performance after the tenth trial per condition (i.e., after approximately 40 trials). This is not surprising, given that participants had to learn which action to perform for 8 instead of 4 gems. Our mixed-effects model confirmed the weaker effect of reward compared to Pilot 2 and Pilot 3 (*b*_ImmvsGreandMean_ = 0.15, 95% HDI [-0.41 0.70]). The effect of reward did not interact with the required action (*b*_ImmvsGreandMean*GovsNoGo_ = -0.01, 95% HDI [-0.20, 0.17]) or the task block (*b*_ImmvsGreandMean*Block_ = 0.001, 95% HDI [-0.11, 0.11]). We also did not observe a statistically significant three-way interaction between reward, required action, and task block (*b*_ImmvsGreandMean*GovsNoGo*Block_ = *b* = 0.04, 95% HDI [-0.08, 0.16]). We observed a statistically significant effect of required action (*b*_GovsNoGo_ = 0.95, 95% HDI [0.57, 1.32]), with more go responses in go trials than no-go trials. Also, again, the effect became stronger over the course of the task, as indicated by a statistically significant interaction between required action and task block (*b*_GovsNoGo*Block_ = 0.39, 95% HDI [0.21, 0.57]). No other statistically significant main effects or interactions were observed.

**Conclusions**

Based on the four pilot studies described above, we expected that the design of Pilot 2 would be most effective in showing the influence of the intertemporal Pavlovian response tendencies on goal-directed behaviour, without frustrating participants with the task difficulty. Therefore, we used the design from Pilot 2 for the full study. We do wish to note that the direction of the hypothesized reward effect was similar across Pilot 2, Pilot 3, and Pilot 4, supporting the robustness of the effect. At the same time, we acknowledge that (i) the marked change in effect size from Pilot 1 to Pilot 2 does not support this robustness, and (ii) small samples were used for the pilot studies, increasing the uncertainty around the observed effects.

**S2. Estimation procedure discount rate**

Each of the 27 choices presented in the Monetary Choice Questionnaire (MCQ; Kirby et al., 1999) corresponded to a discount rate as computed using Mazur’s (1987) hyperbolic discounting model:

*V* = *A* / (1 + *kD*) (1)

with *V* representing the amount of the immediate reward, *A* the amount of the delayed reward, *k* the discount rate (with higher values indicating steeper discounting of future rewards), and *D* the delay until the delivery of the delayed reward. The 27 choices were grouped in nine bins of three choices with the same *k*-value (allowing a maximum of 1% difference between *k*-values in one bin). After participants completed the questionnaire, the nine bins were ranked by *k*-value, from small to large (i.e., patient to impatient). A bin was classified as ‘immediate’ if a participant chose the immediate reward for two or more out of three choices in this bin, and as ‘delayed’ if they chose the delayed reward for two or more choices. If one point existed at which the participant switched from immediate bins to delayed bins, we computed the *k*-value by taking the geometric mean of the last ‘immediate’ bin (i.e., the bin with the highest *k*-value still classified as ‘immediate’) and the first ‘delayed’ bin (i.e., the bin with the lowest *k*-value classified as ‘delayed’ bin). If multiple switch points existed, we chose the switch point that was consistent with the highest number of choices. In case of ties, we took the geometric mean of all equally appropriate *k*-values. If a participant only chose immediate or delayed rewards, we took the *k*-value corresponding to the bin with the highest (*k* = 0.25) or lowest (*k* = 0.00016) *k*-value, respectively.

**S3. Lottery**

At the end of the experiment, participants took part in a lottery in which they had the chance of receiving the outcome they won on one randomly selected trial of the go/no-go task. Their chance of winning the lottery increased as a function of their average response speed in the go/no-go task:

Total chance = 3 + 5 * (1000 – mean RT) / 1000 (2)

This total chance was rounded to 1 decimal place. Next, 100 was divided by this number to create the upper bound for the lottery. Two random numbers were drawn between 1 and this upper bound. If these random numbers were identical, the participant won the lottery. If a participant won the lottery, we randomly selected one trial of the go/no-go task and paid out the reward that the participant won on this trial. If they won the immediate reward, this was paid out as soon as possible on the day of participation. If they won the delayed reward, this was paid out 120 days after participation. If the participant gave an incorrect response on the selected trial, they did not win anything. We used participants’ actual responses to determine their reward, ignoring possible false feedback. Six out of 200 participants won the lottery.

**S4. Susceptibility to Temptation Scale (STS)**

**Table S4.1**

*Susceptibility to Temptation Scale (STS) Items*

| STS1 | I will crave a pleasurable diversion so sharply that I find it increasingly hard to stay on track. |
| --- | --- |
| STS2 | I feel irresistibly drawn to anything interesting, entertaining, or enjoyable. |
| STS3 | I have a hard time postponing pleasurable opportunities as they gradually crop up. |
| STS4 | When an attractive diversion comes my way, I am easily swayed. |
| STS5 | My actions and words satisfy my short-term pleasures rather than my long-term goals. |
| STS6 | I get into jams because I will get entranced by some temporarily delightful activity. |
| STS7 | It takes a lot for me to delay gratification. |
| STS8 | When a task is tedious, again and again I find myself pleasantly daydreaming rather than focusing. |
| STS9 | When a temptation is right before me, the craving can be intense. |
| STS10 | I choose smaller but more immediate pleasures over those larger but more delayed. |
| STS11 | I take on new tasks that seem fun at first without thinking through the repercussions. |

*Note.* Items were scored on a five-point scale (0 = Not true to me, 1 = Not usually true for me, 2 = Sometimes true for me, 3 = Mostly true for me, 4 = True for me). We added one attention check item to the scale, stating “This is an attention check. Please select ‘Not usually true for me’.”. Participants who failed this attention check (*n* = 2) where excluded from data analyses involving the STS.

**S5. Moderators of the Pavlovian bias effect**

**Instrumental Performance**

We examined whether instrumental performance, defined as the proportion of correct responses across the 200 go/no-go trials, moderated the effect of reward (immediate/delayed) on go responding in the go/no-go task. We might see, for instance, that individuals who are uncertain which instrumental response to make towards the cues rely more strongly on Pavlovian response tendencies to make their response than individuals who are certain which instrumental response is correct. It should be noted, however, that lower instrumental performance may also be the *result* of a stronger intertemporal Pavlovian bias. Nevertheless, despite being unable to draw conclusions regarding the direction of this possible association in the current study^[[1]](#footnote-1)^, we explored whether such an association exists.

We reran the main go/no-go task model with instrumental performance as additional, centered, predictor that was allowed to interact with all other predictors. Instrumental performance did not show a two-way interaction with reward (*b* _ImmvsGrandMean*Performance_ = -0.43, 95% HDI [-1.48, 0.60]). We did, however, observe a significant three-way interaction between reward, instrumental performance, and required action (*b* _ImmvsGrandMean*GovsNoGo*Performance_  = -0.69, 95% HDI [-1.26, -0.13]). Figure S5.1 suggests that although in both go trials and no-go trials, lower instrumental performance seemed to be associated with a stronger reward effect, any possible moderation effect may have been stronger in go trials than in no-go trials. Post-hoc tests, however, showed no statistically significant interaction between the reward and instrumental performance in either go trials (*b*_ImmvsGrandMean*Performance_ = -1.12, 95% HDI [-2.44, 0.18]) or no-go trials (*b*_ImmvsGrandMean*Performance_ = 0.27, 95% HDI [-0.74, 1.33]). Thus, although the regression coefficient was larger for go trials than for no-go trials (in line with the visual impression from Figure S5.1), these results do not provide evidence that the reward effect was moderated by instrumental performance in either trial type. We also did not observe a statistically significant main effect of instrumental performance, nor any other interactions involving reward and instrumental performance.

**Figure S5.1**

*Interaction between Reward, Instrumental Performance, and Required Action*

*Note.* The effect of reward on the probability of making a go response, as a function of instrumental performance, separately for go-trials and no-go trials. The figure displays model-based means and 95% Highest Density Intervals. While the model was run with a centered version of the indifference value, and the figure is based on these model predictions, we report the uncentered (raw) performance in the figure legend to aid interpretation of the results.

**Intertemporal impatience**

Next, we explored whether participants’ intertemporal impatience, as observed during the choice titration procedure, moderated the observed effect of reward in the go/no-go task. Although we used preference-matched reward pairs to control for inter-individual differences in subjectively discounted reward value, we might still see that participants who show stronger intertemporal impatience are more susceptible to the intertemporal Pavlovian bias. To examine this, we reran our main go/no-go task model while including the participant-specific centered indifference value (i.e., the immediate reward amount preference-matched to €28 in 120 days), allowing it to interact with all other predictors. There was no statistically significant two-way interaction between reward and indifference value (*b*_ImmvsGrandMean*IV_ = -0.01, 95% HDI [-0.03, 0.01]). However, we did observe a statistically significant three-way interaction between reward, required action, and the indifference value (*b*_DelvsGrandMean*GovsGrandMean*IV_ = 0.01, 95% HDI [0.003, 0.02]). Figure S5.2 suggests that only for no-go trials, increased intertemporal impatience (reflected by a lower indifference value) was associated with a stronger effect of reward on go responding. Follow-up models indeed showed a statistically significant interaction between the indifference value and the reward for no-go trials (*b* = -0.02, 95% HDI [-0.04, -0.003]), but not for go trials (*b* = 0.003, 95% HDI [-0.02, 0.03]). Post-hoc analyses showed that for no-go trials, we observed a significant effect of reward at average indifference values (i.e., average impatience; *b*_ImmvsGrandMean_ = -0.40, 95% HDI [-0.60, -0.21]) and low indifference values (1 *SD* below average, i.e., more impatience; *b*_ImmvsGrandMean_ = -0.22, 95% HDI [-0.36, -0.08]), but not at high indifference values (1 *SD* above average, i.e., less impatience; *b*_ImmvsGrandMean_ = -0.05, 95% HDI [-0.24, 0.15]).

In sum, we observed that for no-go trials, more intertemporal impatience was associated with a stronger effect of reward in the go/no-go task. This suggests that more intertemporally impatient individuals have more difficulties showing goal-directed inhibition in anticipation of an immediate (versus a delayed) reward, compared to more patient individuals. This supports the proposed relevance of the intertemporal Pavlovian bias in intertemporal impatience. Increased intertemporal impatience has been associated with various mental health disorders, such as ADHD, substance use disorders, and borderline personality disorder (Amlung et al., 2019; Lempert et al., 2018; Levin et al., 2018; Levitt et al., 2022). As described in the discussion of the main text, it would therefore be interesting for future research to investigate the role of this intertemporal Pavlovian bias in these mental health problems characterized by intertemporal impatience.

The fact that this moderation was only observed for no-go trials (but not for go trials) may have resulted from a ceiling effect in go responses on go trials, leaving less room for go responses to be increased even further by the anticipated reward and intertemporal impatience. However, it is also possible that intertemporal impatience is predominantly associated with deficits in goal-directed *inhibition*, instead of enhanced goal-directed approach. Since we also observed indications for a stronger main effect of reward in no-go trials than go trials, we further address this issue in the discussion of the main text.

Finally, the direction of the moderation pattern, as evidenced by the post-hoc analyses and as displayed in Figure S5.2, shows that the moderation was not driven by a regression to the mean in discounting estimates. That is, if the choice titration procedure would have resulted in indifference values that were more extreme than participants’ true intertemporal impatience (reflecting measurement error), this could have also resulted in a moderation of the Pavlovian bias by the indifference values. If, for instance, the impatience of participants with low indifference values was not extreme as their indifference value suggested, they would have assigned a higher subjective value to the delayed member of the reward pair that was used in the go/no-go task. As a result, these participants would be expected to show a Pavlovian bias in the opposite direction, with more go responding for delayed compared to immediate rewards. Similarly, if the patience of participants with high indifference values was less extreme than their indifference value suggested, they would be expected to assign a higher subjective value to the immediate member of the reward pair, resulting in a stronger Pavlovian bias effect (in the expected direction). As reported above, however, we observed the opposite moderation pattern, with a *stronger* Pavlovian bias (in the expected direction) in participants with low indifference values, and a *weaker* Pavlovian bias in participants with higher indifference values. Thus, the moderation effect does not appear to be attributable to a regression to the mean in discounting estimates.

**Figure S5.2**

*Interaction between Reward, Intertemporal Impatience, and Required Action*

*Note.* The effect of reward (immediate versus delayed) on the probability of making a go response, as a function of the indifference value derived from the choice titration task that was administered before the go/no-go task, separately for go trials and no-go trials. Lower indifference values reflect more intertemporal impatience. The figure displays model-based means and 95% HDIs. While the model was run with a centered version of the indifference value, and the figure is based on these model predictions, we report the uncentered (raw) indifference values in the figure legend to aid interpretation of the results.

**Reward ratings**

As reported in the main text, despite being preference-matched based on revealed preference, we observed significantly higher attractiveness ratings for the immediate than the delayed rewards. We therefore examined whether the rating difference (immediate – delayed) moderated the Pavlovian bias effect. We reran the main go/no-go model, adding the centered rating difference as additional predictor that was allowed to interact with all other predictors. We observed a statistically significant interaction between the reward effect and the rating difference (*b*_ImmvsGrandMean*_*_RatingDifference_* = 0.01, 95% HDI [0.0004, 0.01]), and subsequently ran post-hoc analyses to examine the reward effect at average, moderately high (0.5 *SD* above average), high (1 *SD* above average), moderately low (0.5 *SD* below average), and low (1 *SD* below average) levels of the centered rating difference. We used these five instead of the more commonly used three (high/average/low) levels of the rating difference because the moderately low (1 *SD* below average) level of the centered rating difference translates to a raw rating difference of zero. This allowed us to examine the effect of reward when the immediate and delayed rewards were rated as equally attractive. The low (2 *SD* below average) level allowed us to examine the effect of reward when the delayed reward was rated higher than the immediate reward, whereas the average, moderately high and high levels allowed us to examine this effect when the immediate reward was rated higher than the delayed reward. Figure S5.3 suggests that although the same pattern of go responding was visible across levels of rating differences—with increased responding in anticipation of immediate compared to delayed rewards—the Pavlovian bias became stronger as the rating difference between the two rewards (with higher ratings for the immediate reward) increased. Supporting this visual impression, the regression coefficients for the effect of reward became larger as the rating difference increased, and only reached statistical significance at high (*b*_ImmvsGrandMean_ = 0.41, 95% HDI [0.16, 0.65]), moderately high (*b* = 0.32, 95% HDI [0.12, 0.50]), and average (*b* = 0.22, 95% HDI [0.05, 0.40]), but not at moderately low (*b* = 0.13, 95% HDI [-0.07, 0.32]), and low (*b* = 0.04, 95% HDI [-0.21, 0.29]) levels of the centered rating difference.

To be able to draw a more detailed comparison between the individuals who rated the immediate and delayed reward equally (with a maximum absolute difference of 5 points on a scale from 0-100^[[2]](#footnote-2)^; *n* = 47), individuals who rated the immediate reward higher than the delayed reward (*n* = 107), and individuals who rated the delayed reward higher than the immediate reward (*n* = 30), we additionally inspected the raw data on the go/no-go task for these three groups of participants. In line with Figure S5.3, Figure S5.4 suggests that the Pavlovian bias effect was strongest in the group of participants who rated the immediate reward higher than the delayed reward, but seems to be in the same direction for participants who rated both rewards equally. However, in contrast to Figure S5.3, the reward effect in the group of participants who rated the delayed reward higher than the immediate rewards seems to have reversed. Note, however, that due to the low sample size of the latter two groups, the confidence intervals in these groups are very wide.

Together, these results suggest that valuation differences (as assessed using the attractiveness ratings) between the immediate and delayed rewards may have contributed to the observed Pavlovian bias effects. Nevertheless, the consistency of the direction of the effect across levels of the moderator suggests that valuation differences are not the only driving force behind the observed effect, leaving room for the hypothesized immediacy-driven conditioned approach response. We return to this issue in the discussion of the main text.

**Figure S5.3**

*Interaction between Reward and Rating Difference*

*Note.* The effect of reward on the probability of making a go response, as a function of the difference in rating of the immediate (I) and delayed (D) reward. The figure displays model-based means and 95% HDIs. While the model was run with a centered version of the rating difference, and the figure is based on these model predictions, we report the raw rating difference in the figure legend to aid interpretation of the results.

**Figure S5.4**

*Pavlovian Bias Effect for different Rewards Rating Groups*

*Note.* Observed behaviour in the go/no-go task for the full sample (panel A), for participants who rated the immediate reward higher than the delayed reward (panel B), for participants who rated the immediate and delayed reward approximately equally high (maximum absolute difference of 5 points on a scale from 0-100; panel C), and for participants who rated the delayed reward higher than the immediate reward (panel D). All panels are based on raw data; the shaded areas display 95% Confidence Intervals.

**S6. Response times and late go responses**

In the main text, we report the effect of reward (immediate / delayed) on the probability of making a go response. Following findings by Luo et al. (2009) that the anticipation of an immediate (versus delayed) reward resulted in faster responses on the Monetary Incentive Delay (MID) task, we also examined the effect of reward on response times in the go/no-go task. We hereby explored whether the reward not only increased the *probability* of making a go response, but also the *speed* with which go responses were made, taking speed as measure of behavioural vigour (in line with Algermissen and den Ouden, 2023; Guitart-Masip et al., 2011, 2012; Scholz et al., 2022; Swart et al., 2017, 2018). To this end, we ran a model that was identical to our main model reported in the main text, except that we used response times (RT) instead of responses (go/no-go) as dependent variable. RTs (in ms) from both correct and incorrect go responses were used. We modelled the RTs with a shifted lognormal family to account for their skewed distribution; the regression coefficients and 95% HDIs below are therefore reported on the log-normal scale. Group means and 95% HDIs are reported on the response scale (in ms) to facilitate interpretation of the effect.

Figure S6.1A-C shows the trial-by-trial mean RTs per condition (Figure S6.1A), the aggregated RTs per condition (Figure S6.1B) and the aggregated RTs per reward (Figure S6.1C). We observed a significant effect of required action (*b*_GoTrialsvsGrandMean_ = 0.01, 95% HDI [0.01, 0.02]), with faster responses for *incorrect* go responses (go responses made on no-go trials, i.e., commission errors; *M* = 414.88, 95% HDI [408.30, 421.58]) than for *correct* go responses (go responses made on go trials; *M* = 423.69, 95% HDI [417.80, 429.66]). This may suggest that incorrect go responses were made more impulsively than correct go responses. There was no significant effect of task block on response times, suggesting that responses did not become significantly faster or slower over the course of the task (*b* = -0.005, 95% HDI [-0.01, 0.001]). We observed no statistically significant effect of reward (*b*_ImmvsGrandMean_ = -0.002, 95% HDI [-0.01, 0.003]), showing that the anticipation of immediate rewards did not result in faster go responses than the anticipation of delayed reward, or vice versa. We did not observe a statistically significant interaction between the reward and task block (*b* = 0.002, 95% HDI [-0.002, 0.005]), showing that the effect of reward did not significantly change throughout the task. The reward and the required action also did not interact (*b* = 0.0004, 95% HDI [-0.005, 0.005]), indicating that the effect of reward was not significantly different for correct compared to incorrect go responses. This contrasts with Figure S6.1B, which seems to suggest that only for incorrect go responses (which, as discussed above, may have been made more impulsively), responses in anticipation of immediate rewards were faster than in anticipation of delayed rewards. This would be consistent with the idea that the anticipation of immediate (versus delayed) rewards increases the vigour with which go responses are made. However, the figure also shows very wide 95% HDIs, reflecting large uncertainty around this effect, possibly partly due the fact that not many incorrect go responses were made (i.e., 15.97% of all responses made in the go/no-go task, and 29.77% of all go responses). We did observe a significant three-way interaction between reward, required action, and block (*b* = -0.003, 95% HDI [-0.006, -0.0004]). Therefore, we examined whether the two-way interaction between reward and required action that is suggested by Figure S6.1B (but that was not statistically significant) changed over the course of the task. Figure S6.2 indeed suggests that this pattern may have only been present in the first two task blocks. However, again, the 95% HDIs around the means were wide. Forming follow-up tests to the statistically significant three-way interaction, we reran our statistical model for each task block separately (excluding the task block predictors). However, none of these follow-up models showed a significant two-way interaction between reward and required action (block 1: *b* = -0.01, 95% HDI [-0.01, 0.0004]; block 2: *b* = 0.003, 95% HDI [-0.005, 0.01]; block 3: *b* = -0.01, 95% HDI [-0.01, 0.0003]; block 4: *b* = -0.001, 95% HDI [-0.01, 0.008]).

In summary, our results do not provide evidence for an intertemporal Pavlovian bias on response vigour, as measured through response speed. While a visual inspection of response times suggests that for go responses made on no-go trials (i.e., commission errors), the anticipation of immediate rewards increased response speed compared to the anticipation of delayed rewards—particularly in the first half of the task—we did not observe any statistically significant effects supporting this visual impression.

**Figure S6.1**

*Response Time Effects*

**B**

**A**

**C**

*Note.* Raw response times (RTs; with 95% Confidence IntervaIs). Panel A: Average trial-by-trial response times (RTs) per condition. Panel B: Average RTs, aggregated per condition. Panel C: Average RTs, aggregated over go and no-go trials.

**Figure S6.2**

*Reward, Required Action, and Task Block Interaction*

*Note.* Mean model-based response times (RTs; with 95% HDIs) per reward, required action, and task block. The RT values on the y-axis were back-transformed from the lognormal scale to the response scale. Note that RTs on no-go trials were incorrect go responses (i.e., commission errors).

**Late go responses**

As preregistered, we also examined the effect of reward on late go responses. First, we explored whether the probability of making a late go response was influenced by the reward. Since the anticipation of immediate rewards increased the probability of making a go response *within* the response window, we may also see a lower probability of *late* responses for immediate (versus delayed) rewards. To examine this, we created a subset of the data that only included go responses made on go trials, either in time (within 600 ms) or too late (after the response deadline but before the start of the next trial, i.e., between 600-2300 ms after cue onset). Of these responses, 11.23% were late responses. We excluded go responses made on no-go trials to reduce the chance of including late go responses that were made without the intention of making a go response, but were due to, e.g., accidental or random button pressing. We modelled whether or not a response was late (yes / no) as a function of reward (immediate/delayed), task block (centered) and their interaction as fixed effects and as random effects varying over participants (also including the random intercepts and correlations), using a Bernoulli model family to account for the binary response variable. We did not observe a statistically significant effect of reward (*b*_DelayedvsGrandMean_ = 0.02, 95% HDI [-0.08, 0.13]), indicating that the probability of making a late go response on go trials was not influenced by the anticipated reward. Thus, although the anticipation of immediate (versus delayed) rewards increased the probability of making a go response *within* the response window (as reported in the main text), this was not accompanied by a decreased probability of making *late* responses. We did observe a statistically significant effect of task block (*b* = -0.48, 95% HDI [-0.58, -0.36]), with a decreasing probability of late responses over the course of the task. This may reflect the generally increasing accuracy over the course of the task, as participants learned to make go responses within the response window. The effect of task block did not interact with reward, indicating that the effect of task block was not significantly different for immediate compared to delayed rewards (*b* = 0.03, 95% HDI [-0.04, 0.09]).

Second, we investigated the effect of reward on the RTs of late go responses only, hereby examining the possibility that, although these responses were too slow, they may have still been faster in anticipation for immediate than delayed reward. This may be especially relevant at the start of the task, during which we observed a higher probability of late go responses compared to the end of the task (likely because participants were still learning to give the correct response in time). We analysed the RTs (modelled using a shifted lognormal distribution) as a function of reward (immediate/delayed), task block (centered), and their interaction as fixed effects and as random effects varying over participants (also including the random intercepts and correlations). We observed no significant effect of reward (*b*_DelayedvsGrandMean_ = 0.01, 95% HDI [-0.03, 0.06]), indicating that the reward (immediate/delayed) did not influence RTs of late responses. We also did not observe any significant effect of task block (*b* = 0.00003, 95% HDI [-0.04, 0.04]) or interaction between reward and task block (*b* = -0.01, 95% HDI [-0.05, 0.03]), indicating that late RTs did not become significantly faster or slower over the course of the task, and that this did not differ between immediate and delayed rewards.

In sum, we did not observe any statistically significant effect of reward on the probability of making a late go response, or on the RTs of late go responses.

**S7. Individual differences in behaviour and model fit**

As displayed in Figure S7.1, we observed substantial inter-individual differences in the effect of reward (immediate/delayed) on go responding. While 51% of participants showed an effect in the expected direction (i.e., a regression coefficient of 0.1 or higher), 15% showed no or a negligible effect (-0.1 < *b* < 0.1), and 34% showed the opposite effect (*b* ≤ -0.1). Although the effect estimates are not extreme in either direction, the fact that a substantial number of participants showed the opposite effect is somewhat surprising. Possible explanations may involve the statistically significant moderators of the reward effect. As reported in detail in S5, we observed the reward effect to be moderated by inter-individual variability in the difference in attractiveness ratings between the immediate and delayed reward, as well as, for the no-go trials, inter-individual variability in intertemporal impatience. Post-hoc analyses showed that higher attractiveness ratings of the immediate (compared to the delayed) reward, and, for the no-go trials, stronger intertemporal impatience, were associated with a stronger reward effect. It should be noted that the model-based post-hoc analyses did not show a reversed reward effect for individuals who rated the delayed reward higher than the immediate reward, or for individuals who showed little intertemporal impatience, as the regression coefficient in these groups was still positive. Nevertheless, the effect in these groups was substantially weaker and not statistically significant. This increases the likelihood that at least some of the participants in these groups showed a pattern of responding that was in the opposite direction of what we hypothesized. Moreover, Figure S5.4, which displays raw data instead of model-based estimates, suggests that participants who rated the delayed reward higher than the immediate reward did show a reversed reward effect, with more go responding in anticipation of delayed rewards. We remain careful in drawing conclusions from Figure S5.4, as the confidence intervals were wide and we did not run statistical tests. Nevertheless, this pattern points towards the possible role of reward valuation in explaining the reversed reward effect. Similarly, it is tempting to speculate that at least some of the very patient participants (i.e., with high indifference values), who did not show a statistically significant reward effect on no-go trials, may have shown a pattern of responding that was opposite to what we predicted.

**Individual differences in RL Model Fit**

As displayed in Figure 3E in the main text, we also observed substantial inter-individual differences in the reinforcement learning model that showed the best fit to the go/no-go data. A possible explanation for this observation is that participants may differ in the mechanisms that drive the observed reward effect on go responding. For some participants, this effect may be driven by a cue-response bias (M3); for others, it may be driven by a learning bias (M4); and yet for others, it may be driven by a combination of both (M5). Moreover, we observed that for some participants, the best-fitting model did not include any Pavlovian bias parameters (i.e., M0-M2), which may result from the inter-individual differences in the behavioural reward effect discussed above. Figure S7.2 indeed shows that, for the group of participants who showed no reward effect, the relatively simpler models (mostly M2) tended to be the best-fitting model more often than models that included Pavlovian bias parameters. For participants who showed either the expected reward effect (with increased go responding in anticipation of immediate versus delayed rewards) or the opposite effect (increased go responding in anticipation of delayed versus immediate rewards), the more complex models showed a higher proportion of being the best-fitting model, with the highest proportion for M4 in the expected effect group and M5 in the opposite effect group.

The observation that models M3-5 capture both the expected, as well as the opposite reward effect, was supported by statistically significant positive correlations between the Pavlovian bias parameters in these models and the behavioural reward effect estimate (see Figure S7.3). A larger absolute π parameter was associated with a stronger behavioural reward effect in the expected direction (M3: *r* = .42, *p* < .001; M5: *r* = .31, *p* < .001). Similarly, in M4, a higher bias-congruent learning rate (α_0_) was associated with a stronger behavioural reward effect in the expected direction (*r* = .25, *p* = .001). This points towards an association between the absolute strength of the Pavlovian bias effect and the parameters that aim to capture this effect, regardless of the direction of the effect. We did not observe this relation in M5 (*r* = .12, *p* = .130), nor did we find a correlation between the bias-incongruent learning rate (α_1_) and the reward estimate in M4 and M5 (M4: *r* = .0004, *p* = .995; M5; *r* = .09, *p* = .223). These results suggest that the cue-response (π) parameter can capture both the expected and opposite reward effect, and that in M5, this parameter does this better than the learning rate parameters. In M4, it seems to be the bias-congruent learning rate that captures this effect. We do wish to note, however, that the suboptimal parameter recovery of M4 and M5 (see S8) warrants us to remain careful in drawing strong conclusions regarding individual model parameters of these models.

Finally, we acknowledge that M0 and, to a lesser extent, M2, even were the best-fitting models for a substantial proportion of participant who did show the expected or opposite reward effect (i.e., this was not only the case for participants who did not show any reward effect). Thus, at least for a subset of these participants, their behaviour is best-fitted by a simpler Rescorla-Wagner model.

**Figure S7.1**

*Individual Differences in Reward Effect*

*Note.* Density plot showing the distribution of the effect estimate of reward (immediate versus delayed) on go responding across participants. The reward estimate is reported on log odds scale. A positive estimate indicates more go responding in anticipation of immediate (versus) delayed rewards; a negative estimate indicates the opposite effect. The dashed blue, vertical line indicates the mean reward estimate across participants (*b* = 0.17).

**Figure S7.2**

*Association between Reward Effect and Model Frequency*

*Note.* Bar plot displaying the relation between the behavioural reward effect and the frequency with which each model formed the best-fitting RL model. Participant-level reward effect estimates were extracted from our main mixed-effects model, and participants were subsequently divided in one of three groups: a group of participants who showed the expected reward effect (i.e., increased go responding in anticipation of immediate versus delayed rewards; *n* = 94), a group of participants who did not show a reward effect (defined as a reward estimate between -0.1 and 0.1; *n* = 28), and a group of participants who showed the opposite effect (i.e., increased go responding in anticipation of delayed versus immediate rewards; *n* = 62). For each of the three groups separately, we computed the proportion of participants for whom each RL model was the best-fitting model (*p*[bestfit]). For instance, for 22% of the 94 participants in the *Expected Effect* group, M0 was the best-fitting model.

**Figure S7.3**

*Relation Reward Effect Estimate and Pavlovian Bias Parameters*

*Note.* Association between the estimate (i.e., the regression coefficient) of the reward effect on go responding and the RL model parameters in M3-M5 that aim to capture this effect. The model parameters include the cue-response bias parameter in M3 (panel A) and M5 (panel D), the bias-congruent learning rate (in M4, panel B, and M5, panel E), and the bias-incongruent learning rate (in M4, panel C, and M5, panel F).

**S8. Parameter and model recovery**

Comparing the model fit of the five fitted reinforcement learning models showed no clear winner between the cue-response bias model (M3), the learning bias model (M4), and the model combining cue-response bias and learning bias (M5). We therefore conducted a model validation for all three models, using parameter recovery, model recovery, and posterior predictive checks (as recommended by Wilson & Collins, 2019). The results of the posterior predictive checks are reported in the main text. Here, we report in detail the results of the parameter and model recovery.

**Parameter Recovery**

For each model (M3-M5), we simulated a go/no-go dataset with 184 participants and 200 trials (similar to the observed dataset). The parameter values used for these simulations were obtained by randomly sampling from the priors used in the MAP estimation, or, for those parameters for which we did not use a prior in the MAP estimation, by sampling from a uniform distribution (see Table S8.1; see further below for the description and results of an alternative approach in which we use the observed best-fitting parameters to generate data). We subsequently fitted each model to its respective dataset, and checked whether we could recover the parameter values that were used to generate the data (i.e., the data-generating parameters). Parameter recovery was quantified as the Pearson correlation between the data-generating parameter values and the parameter values estimated after running the model on these simulated data (i.e., the recovered parameters). A higher correlation indicates better parameter recovery. Figures S8.1-S8.3 show scatterplots of the data-generating and recovered parameters for M3-M5, visualizing the parameter recovery for each of these models.

All models showed good to excellent parameter recovery of the irreducible noise and go bias parameters. Recovery of the cue-response bias parameter was satisfactory in M3 and M5, but slightly better for M3. Recovery of the learning rate and inverse temperature parameters was not as good: Correlations between data-generating and recovered values were generally lower for these parameters, and failed to reach statistical significance for the bias-congruent learning rate in M4. Finally, across the three models, we observed some correlations between recovered parameters, indicating that these parameters may be trading off against each other. This could suggest that these parameters have comparable effects on behaviour, which, for instance, is not implausible for the two learning rate parameters in M4 (Daw, 2011; Wilson & Collins, 2019). It should be noted, however, that the observed correlations among the recovered parameters did not exist among the data-generating parameters, suggesting that they may have also been introduced by the recovery process (Wilson & Collins, 2019).

In sum, recovery of the irreducible noise, go bias, and cue-response bias parameters was satisfactory for all models; recovery of the other parameters was less consistent across models. It should be noted, however, that we adopted a relatively conservative approach by examining parameter recovery for the full range of plausible parameter values. Rerunning the recovery for the restricted range of observed best-fitting parameters resulted in improved recovery of the learning rate and inverse temperature parameters; results are reported in detail below. We also examined whether using an alternative model parametrization that is more similar to that originally used by Swart et al. (2017, 2018) and that uses a non-linear transformation to avoid a hard boundary condition, would improve parameter recovery for M4 (see further below for details). Since this did not substantially improve parameter recovery, we decided to retain the original model parametrization.

**Table S8.1**

*Sampling Distributions for Parameters in Data Simulations*

| Model | Sampling distributions |
| --- | --- |
| M3 | α ~ Uniform(0, 1), τ ~ Gamma(3, 0.3), ξ ~ Uniform(0, 1), *b* ~ Gaussian(0, 1),  π ~ Gaussian(0,1) |
| M4 | α_0_ ~ Uniform(0, 1), α_1_ ~ Uniform(0, 1), τ ~ Gamma(3, 0.3), ξ ~ Uniform(0, 1), *b* ~ Gaussian(0, 1) |
| M5 | α_0_ ~ Uniform(0, 1), α_1_ ~ Uniform(0, 1), τ ~ Gamma(3, 0.3), ξ ~ Uniform(0, 1), *b* ~ Gaussian(0, 1), π ~ Gaussian(0,1) |

*Note.* Data were simulated for each of the three models separately. The parameters used for the simulations were obtained by randomly sampling from the distributions listed in the table.

**Figure S8.1**

*Parameter Recovery M3*

*Note****.*** Parameter recovery of M3 (cue-response bias model). Scatterplots represent the relation between the parameters used to simulate the data (i.e., the data-generating parameters), and the parameters obtained when fitting M3 to the simulated data (i.e., the recovered parameters). The data-generating parameters were obtained by randomly sampling from the distributions listed in Table S8.1. Parameter recovery of the irreducible noise parameter ξ (*r* = .86, *p* < .001), the go bias parameter *b* (*r* = .73, *p* < .001), and the cue-response bias parameter π (*r* = .69, *p* < .001) was good to excellent. Parameter recovery of the learning rate parameter α (*r* = .43, *p* < .001) and the inverse temperature parameter τ (*r* = .19, *p* = .011), was not as good, although the correlations were still positive and statistically significant. We observed statistically significant correlations between the recovered α and the recovered ξ parameter (*r* = .17, *p* = .023), and between the recovered α and the recovered τ parameter (*r* = .20, *p* = .005), which may indicate that parameters were trading off against each other to a certain extent. We did not observe any correlations between the data-generating parameters, suggesting that the correlation between the recovered parameters may have been introduced by the recovery process (Wilson & Collins, 2019).

**Figure S8.2**

*Parameter Recovery M4*

**

*Note****.*** Parameter recovery of M4 (learning bias model). Similar to M3, parameter recovery of the irreducible noise parameter ξ (*r* = .88, *p* < .001) and the go bias parameter *b* (*r* = .78, *p* < .001) was good to excellent. Again, parameter recovery of the inverse temperature parameter τ was not as good (*r* = .24, *p* = .001), but still positive and statistically significant. The bias-congruent learning rate parameter α_0_ was not recovered (*r* = .03, *p* = .675). Parameter recovery of the bias-incongruent learning rate parameter α_1_ was better (*r* = .36, *p* < .001). The recovered learning rate parameters α_0_ and α_1_ were significantly correlated (*r* = .23, *p* = .002), as were the recovered α_0_ and *b* (*r* = -.18, *p* = .014), indicating that these parameters may have been trading off against each other. The data-generating versions of these parameters were not significantly correlated, suggesting that the correlations between the recovered parameters may have been introduced by the recovery process. We did observe a statistically significant correlation between the data-generating τ and *b* parameters (*r* = .16, *p* = .029).

**Figure S8.3**

*Parameter Recovery M5*

*Note****.*** Parameter recovery of M5 (combined cue-response bias and learning bias model). Similar to M3 and M4, parameter recovery of the irreducible noise parameter ξ (*r* = .94, *p* < .001) and the go bias parameter *b* (*r* = .76, *p* < .001) were good to excellent. Parameter recovery of the cue-response bias parameter π (*r* = .61, *p* < .001) was not as good as in M3, but still moderately positive and statistically significant. Similar to M3 and M4, the recovery of the inverse temperature τ was not satisfactory, albeit positive and statistically significant (*r* = .27, *p* < .001). Similar to M4, recovery of the bias-incongruent learning rate parameter α_1_ (*r* = .32, *p* < .001) was better than recovery of the bias-congruent learning rate parameter α_0_ (*r* = .24, *p* = .001), but this time, both correlations were statistically significant. The recovered α_0_ and τ were significantly correlated (*r* = .22, *p* = .003), indicating that these parameters may be trading off against each other. The data-generating version of these parameters did not significantly correlate, suggesting that the correlation between the recovered parameters may have been introduced by the recovery process. The data-generating parameters did include a correlation between α_1_ and *b* (*r* = .16, p = .031) that did not exist among the recovered parameters.

**Model Recovery**

As a second form of model validation, we examined whether we could correctly identify the data-generating model using model comparison. We used the same simulated datasets as for the parameter recovery (generated using M3, M4, and M5, respectively), and subsequently fitted each of the three models to each of the three datasets (resulting in 9 model fits). Perfect model recovery would imply that the model that was used to generate the data fitted the simulated data best, indicating that the models are fully distinguishable using model comparison. We indeed observed M3 to be the best-fitted model when the data were generated by M3 (Figure S8.4A). However, when the data were generated by M4 (Figure S8.4B) and M5 (Figure S8.5C), M3 again showed the best fit — although the differences in median AIC values were small. Following Danwitz et al. (2022), we also created a confusion matrix that shows, for each data-generating model, the proportion of simulated participants for whom each of the three models fitted best (Table S8.2). In the case of perfect model recovery, the matrix would be 1 on the diagonal, and 0 everywhere else. As can be seen in the table, this is not the case, indicating less-than-perfect model recovery. Nevertheless, for the majority of participants, M3 fitted best to data generated by M3, in line with Figure S8.4A. Model recovery for M4 was not as good, with a similar proportion of participant simulated by M4 for whom M3 and M4 fitted best. For M5, model recovery was particularly problematic, as for only 11% of the participants simulated by M5, M5 was the best-fitting model. Given that M5 is a combination of M3 and M4, we do not deem it too surprising that this model was not well distinguishable from the other two models. Moreover, the higher parsimony of M3 and M4 compared to M5 increased the chance that these models showed lower AIC values compared to M5. It is of note, however, that M4 showed a worse fit to data generated by M5 than M3, suggesting that the Pavlovian bias parameter has a relatively strong influence on the model recovery process.

In sum, we could correctly identify M3 as best-fitting model when the data were generated using M3, showing successful model recovery for M3. In contrast, model recovery for M4, and especially M5, was not successful. Again, however, we should note that when simulating data using the observed best-fitting range of parameter values instead of the full range of parameter values results, model recovery of M4 and M5 improved, as described in detail below. Nevertheless, the pattern remained unchanged, with the best model recovery for M3.

**Figure S8.4**

*Model Recovery*

*Note****.*** Model recovery of M3, M4, and M5. We used the same three simulated datasets as for the parameter recovery. Each model was subsequently fitted to each of the three datasets (resulting in 9 model fits). If the models were fully distinguishable using model comparison, one would expect the model that generated the data to fit the simulated data best. Model fit is displayed using median AIC values across participants.

**Table S8.2**

*Confusion Matrix Model Recovery*

|  |  | Best-fitting model | | |
| --- | --- | --- | --- | --- |
|  |  | M3 | M4 | M5 |
| Data-generating model | M3 | .71 | .22 | .07 |
|  | M4 | .47 | .42 | .11 |
|  | M5 | .65 | .25 | .10 |

*Note.* Confusion matrix indicating, for each data-generating model, the proportion of simulated participants for whom each of the three models fitted best. For instance, for 71% of the participants simulated by M3, M3 was the best-fitting model. Model fit was assessed using AIC.

**Recovery with Observed Best-Fitting Parameter Values**

For the parameter and model recovery procedures reported above, we obtained data-generating parameter values by randomly sampling from the prior distributions used for the MAP estimation, or, for those parameters for which we did not use priors, from a uniform distribution. This is a relatively conservative approach, because it examines whether, across the *full range* of plausible parameter values (with plausible being defined by the sampling distributions), the parameters could be recovered. An alternative, somewhat less conservative approach also found in the literature involves examining whether, across the range of *actually* *observed per-participant best-fitting* parameter values, the parameters are successfully recovered (Wilson & Collins, 2019). To gain more insight into the range of parameter values in which parameter and model recovery was more or less successful, we reran our recovery procedure for all three models using the specific range of actually observed per-participant best-fitting parameter values to generate data. Perhaps not too surprisingly, using this more restricted range resulted in improved parameter recovery, in particular for the learning rates and inverse temperature. Thus, whereas parameter recovery for these parameters was not always satisfactory across the full range of plausible parameter values, it was better in the specific range of observed best-fitting parameter values. An overview of correlations between the data-generating and recovered parameters for all three models can be found in Table S8.3. An overview of the *model* recovery results can be found in Figure S8.5 and Table S8.4. Consistent with the improved parameter recovery for the learning rates, the improvement in model recovery seemed to be more marked for those models that included two learning rates parameters (i.e., M4 and M5). Nevertheless, consistent with our original recovery procedure, Figure S8.5 and Table S8.4 point towards the most successful recovery for M3 compared to M4 and, in particular, M5.

In sum, these results suggest that parameter and model recovery was better for the range of parameter values that we observed to be best-fitting to our data, than for the full range of parameter values, particularly for the models that included two learning rate parameters (M4 and M5). As argued by Wilson and Collins (2019), successful recovery for the range of observed parameter is more essential than successful recovery for the full range of parameter values. Both recovery approaches, importantly, show most successful recovery for M3.

**Table S8.3**

*Parameter Recovery Results with Observed Best-Fitting Parameters*

| Model | Parameter | *r* | *p* |
| --- | --- | --- | --- |
| M3 | α | .75 | < .001 |
|  | τ | .51 | < .001 |
|  | ξ | .86 | < .001 |
|  | *b* | .90 | < .001 |
|  | π | .85 | < .001 |
| M4 | α_0_ | .42 | < .001 |
|  | α_1_ | .68 | < .001 |
|  | τ | .54 | < .001 |
|  | ξ | .89 | < .001 |
|  | *b* | .87 | < .001 |
| M5 | α_0_ | .50 | < .001 |
|  | α_1_ | .42 | < .001 |
|  | τ | .36 | < .001 |
|  | ξ | .92 | < .001 |
|  | *b* | .89 | < .001 |
|  | π | .69 | < .001 |

*Note.* Correlations between the data-generating and recovered parameters for M3, M4, and M5. For this recovery approach, we used the per-participant best-fitting parameter values as data-generating parameter values.

**Figure S8.5**

*Model Recovery with Observed Best-Fitting Parameters*

*Note****.*** Model recovery of M3, M4, and M5 when using the per-participant best-fitting parameter values as data-generating parameter values. We used the same three simulated datasets as for the parameter recovery. Each model was subsequently fitted to each of the three datasets (resulting in 9 model fits). If the models were fully distinguishable using model comparison, one would expect the model that generated the data to fit the simulated data best. Model fit is displayed using median AIC values across participants.

**Table S8.4**

*Confusion Matrix Model Recovery with Observed Best-Fitting Parameters*

|  |  | Best-fitting model | | |
| --- | --- | --- | --- | --- |
|  |  | M3 | M4 | M5 |
| Data-generating model | M3 | .60 | .30 | .10 |
|  | M4 | .41 | .47 | .13 |
|  | M5 | .52 | .30 | .30 |

*Note.* Confusion matrix indicating, for each data-generating model, the proportion of simulated participants for whom each of the three models fitted best. For instance, for 60% of the participants simulated by M3, M3 was the best-fitting model (percentages may not add up to exactly 100% due to rounding). Model fit was assessed using AIC. For this recovery approach, we used the per-participant best-fitting parameter values as data-generating parameter values.

**Alternative Parametrization M4**

In line with a suggestion from a reviewer, we also examined whether parameter recovery for M4 could be improved by using an alternative model parametrization, and by using a non-linear transformation to avoid a hard boundary condition. The model specification was as follows:

$= inv.logit\left( {}_{\mathrm{int}}+ {}_{\mathrm{slp}}*congruency \right)$ (3)

with congruency being 1 for bias-congruent conditions (i.e., go responses followed by an immediate reward and no-go responses followed by a delayed reward) and -1 for bias-incongruent conditions (i.e., the remaining conditions). The α_int_ and α_slp_ parameters were constrained between -10 and 10. Fitting the model resulted in a median AIC value of 163.36, highly similar to the original model (median AIC = 164.07). For parameter recovery, we again simulated a go/no-go dataset with 184 participants and 200 trials. We obtained data-generating parameter values for α_int_ and α_slp_ by randomly sampling from a uniform distribution between -10 and 10; values for all other parameters were obtained in the same manner as for the original model. In contrast to our original model, both learning rate parameters showed statistically significant correlations between the data-generating and recovered parameter values (α_int_: *r* = .17, *p* = .021; α_slp_: *r* = .28, *p* = < .001). However, the correlations remained weak, indicating suboptimal recovery, consistent with the original model (original model: α_0_: *r* = .03, *p* = .675; α_1_: *r* = .36, *p* = < .001). Moreover, the correlation coefficient for the α_slp_ was smaller than that of α_1_ of the original model, as was the case for the ξ and *b* parameters (original model: ξ: *r* = .88, *p* < .001, *b*: *r* = .78, *p* < .001, τ: *r* = .24, *p* = .001; adjusted model: ξ: *r* = .78, *p* < .001, *b*: *r* = .71, *p* < .001, τ: *r* = .25, *p* = .001). Thus, overall, the adjusted model parametrization did not seem to result in a substantial improvement compared to the original parametrization, and we therefore decided to retain our original model parametrization.

**S9. Cue ratings**

We examined whether there were any pre-existing individual differences in ratings of the four cues (i.e., the gems), and whether these preferences changed over the course of the experiment. Figure S9.1 shows the ratings of the cues before and after the go/no-go task. We ran a model with rating as dependent variable, stimulus, time (pre/post) and their interaction as fixed effects, and a random intercept, stimulus and time as random slopes, and random correlations as random effects. We tested all possible pairwise comparisons between stimuli using the package emmeans (Lenth, 2019). Before the task, the cyan pentagon was liked significantly better than the red triangle (*b* = 11.49, 95% HDI [6.91, 16.07]) and the purple diamond (*b* = 15.41, 95% HDI [10.43, 20.23]). The gold pentagon was liked significantly better than the red triangle (*b* = 8.50, 95% HDI [3.53, 13.15]) and the purple diamond (*b* = 12.42, 95% HDI [7.53, 17.52]). There was no significant difference in ratings of the cyan and gold pentagon (*b* = 2.99, 95% HDI [-1.68, 7.10]), and of the red triangle and purple diamond (*b* = 3.72, -0.03, 7.78]). By randomly assigning cues to conditions in the go/no-go task, we prevented these pre-existing preferences from confounding the Pavlovian bias effect in the go/no-go task. We observed no significant effect of time (*b*_PostvsGrandMean_ = 0.53, 95% HDI [-0.37, 1.5]), indicating that, averaged across stimuli, the ratings did not significantly change over the course of the experiment. We also observed no interactions between time and any of the pairwise stimulus comparisons, indicating that none of these comparisons changed significantly over the course of the task.

**Figure S9.1**

*Cue Ratings*

*Note.* Ratings of the cues before (Pre) and after (Post) the go/no-go task. The figure is based on raw data. Participants were asked to indicate how attractive they found each of the cues, using a visual analogue scale ranging from 0 (very unattractive) to 100 (very attractive).

**Effect of go/no-go task on ratings**

Research on go/no-go trainings has observed that the required action (go/no-go) associated with food items in a go/no-go task influences the subjective ratings of these items, with the most robust support for a devaluation of items paired with no-go responses (Veling et al., 2022). Although not of interest to our research question, we explored whether we find similar results for our more abstract gem cues, and whether the reward (immediate/delayed) associated with the cue influenced the ratings. We ran a model with ratings as dependent variable, reward (immediate/delayed), required action (go/no-go), time (pre/post), and their interaction as fixed effects, and reward, required action, time and their interactions (except the three-way interaction) as random effects varying over participants (including random intercepts and correlations). We observed a significant effect of required action (*b* = 2.79, 95% HDI [1.33, 4.32]), with higher ratings for go-cues (M_Go_ = 62.10, 95% HDI [60.00, 64.20]) than no-go cues (*M*_No-Go_ = 56.50, 95% HDI [54.20, 58.90]). Crucially, this effect interacted with time (*b*_GovsGrandMean*PostvsGrandMean_ = 3.44, 95% HDI [2.49, 4.41]): While there was no effect of required action before the go/no-go task (*M*_Go_ = 58.10, 95% HDI [55.70, 60.70]); *M*_No-Go_ = 59.40, 95% HDI [56.90, 61.80]; *b_GovsNo-Go_* = -1.30, 95% HDI [-4.38, 1.95]), go-cues received significantly higher ratings than no-go cues after the go/no-go task (*M*_Go_ = 66.00, 95% HDI [63.60, 68.40]); *M*_No-Go_ = 53.60, 95% HDI [50.70, 56.50], *b*_GovsNoGo_ = 12.50, 95% HDI [8.66, 16.40]). These results extend previous work by showing that also for abstract cues such as gems, consistently giving go- or no-go responses to the cues can alter their subjective ratings. It should be noted, however, that because our experiment did not include a control condition with untrained items, we cannot conclude whether this effect was driven by an increased value of go-cues or a decreased value of no-go cues.

We did not observe a significant effect of reward (*b*_ImmvsGrandMean_ = 0.42, 95% HDI [-1.01, 1.86]), nor a reward by time interaction (*b*_ImmvsGrandMean*PostvsGrandMean_ = -0.53, 95% HDI [-1.60, 0.58]), showing that the reward assigned to the cue did not change the ratings of the cues. The absence of an effect of the reward cue ratings could indicate that the reward (immediate versus delayed) associated with the cue indeed does not influence the rated attractiveness of the cue. At the same time, it supports the idea that the rewards were preference-matched (in contrast to what the reward ratings suggested), thereby not resulting in any statistically significant difference in ratings.

We did not observe any other statistically significant main effect or interactions in this model.

**References**

Algermissen, J., & den Ouden, H. E. M. (2023). Goal-directed recruitment of Pavlovian biases through selective visual attention. *Journal of Experimental Psychology: General*. https://doi.org/10.1037/xge0001425

Amlung, M., Marsden, E., Holshausen, K., Morris, V., Patel, H., Vedelago, L., Naish, K. R., Reed, D. D., & McCabe, R. E. (2019). Delay discounting as a transdiagnostic process in psychiatric disorders: A meta-analysis. *JAMA Psychiatry*, *76*(11), 1176–1186. https://doi.org/10.1001/jamapsychiatry.2019.2102

Cartoni, E., Moretta, T., Puglisi-Allegra, S., Cabib, S., & Baldassarre, G. (2015). The relationship between specific Pavlovian instrumental transfer and instrumental reward probability. *Frontiers in Psychology*, *6*, Article 1697. https://doi.org/10.3389/fpsyg.2015.01697

Danwitz, L., Mathar, D., Smith, E., Tuzsus, D., & Peters, J. (2022). Parameter and model recovery of reinforcement learning models for restless bandit droblems. *Computational Brain and Behavior*, *5*(4), 547–563. https://doi.org/10.1007/s42113-022-00139-0

Daw, N. D. (2011). Trial-by-trial data analysis using computational models. *Decision Making, Affect, and Learning: Attention and Performance XXIII*, 1–26. https://doi.org/10.1093/acprof:oso/9780199600434.003.0001

Guitart-Masip, M., Chowdhury, R., Sharot, T., Dayan, P., Duzel, E., & Dolan, R. J. (2012). Action controls dopaminergic enhancement of reward representations. *Proceedings of the National Academy of Sciences of the United States of America*, *109*(19), 7511–7516. https://doi.org/10.1073/pnas.1202229109

Guitart-Masip, M., Fuentemilla, L., Bach, D. R., Huys, Q. J. M., Dayan, P., Dolan, R. J., & Duzel, E. (2011). Action dominates valence in anticipatory representations in the human striatum and dopaminergic midbrain. *Journal of Neuroscience*, *31*(21), 7867–7875. https://doi.org/10.1523/JNEUROSCI.6376-10.2011

Kirby, K. N., Petry, N. M., & Bickel, W. K. (1999). Heroin addicts have higher discount rates for delayed rewards than non-drug-using controls. *Journal of Experimental Psychology: General*, *128*(1), 78–87. https://doi.org/10.1037//0096-3445.128.1.78

Lempert, K. M., Steinglass, J. E., Pinto, A., Kable, J. W., & Simpson, H. B. (2018). Can delay discounting deliver on the promise of RDoC? *Psychological Medicine*, *49*(2), 190–199. https://doi.org/10.1017/S0033291718001770

Lenth, R. (2019). *emmeans: Estimated marginal means*. (Version 1.8.1.1). [Computer software].

Levin, M. E., Haeger, J., Ong, C. W., & Twohig, M. P. (2018). An examination of the transdiagnostic role of delay discounting in psychological inflexibility and mental health problems. *Psychological Record*, *68*(2), 201–210. https://doi.org/10.1007/s40732-018-0281-4

Levitt, E. E., Oshri, A., Amlung, M., Ray, L. A., Sanchez-Roige, S., Palmer, A. A., & MacKillop, J. (2022). Evaluation of delay discounting as a transdiagnostic research domain criteria indicator in 1388 general community adults. *Psychological Medicine*. https://doi.org/10.1017/S0033291721005110

Luo, S., Ainslie, G., Giragosian, L., & Monterosso, J. R. (2009). Behavioral and neural evidence of incentive bias for immediate rewards relative to preference-matched delayed rewards. *Journal of Neuroscience*, *29*(47), 14820–14827. https://doi.org/10.1523/JNEUROSCI.4261-09.2009

Mazur, J. E. (1987). An adjusting procedure for studying delayed reinforcement. In M. L. Commons, J. E. Mazur, J. A. Nevin, & H. Rachlin (Eds.), *Quantitative analyses of behavior: Vol 5. The effect of delay and of intervening events on reinforcement.* (pp. 55–73). Lawrence Erlbaum Associates.

Scholz, V., Hook, R. W., Kandroodi, M. R., Algermissen, J., Ioannidis, K., Christmas, D., Valle, S., Robbins, T. W., Grant, J. E., Chamberlain, S. R., & den Ouden, H. E. M. (2022). Cortical dopamine reduces the impact of motivational biases governing automated behaviour. *Neuropsychopharmacology*, *47*, 1503–1512. https://doi.org/10.1038/s41386-022-01291-8

Swart, J. C., Cook, J. L., Geurts, D. E., Frank, M. J., Cools, R., & den Ouden, H. E. M. (2017). Catecholaminergic challenge uncovers distinct Pavlovian and instrumental mechanisms of motivated (in)action. *eLife*, 6:e22169. https://doi.org/10.7554/eLife.22169.001

Swart, J. C., Frank, M. J., Määttä, J. I., Jensen, O., Cools, R., & den Ouden, H. E. M. (2018). Frontal network dynamics reflect neurocomputational mechanisms for reducing maladaptive biases in motivated action. *PLoS Biology*, *16*(10), 1–25. https://doi.org/10.1371/journal.pbio.2005979

Veling, H., Becker, D., Liu, H., Quandt, J., & Holland, R. W. (2022). How go / no-go training changes behavior: A value-based decision-making perspective. *Current Opinion in Behavioral Sciences*, *47*, 101206. https://doi.org/10.1016/j.cobeha.2022.101206

Wilson, R. C., & Collins, A. G. E. (2019). Ten simple rules for the computational modeling of behavioral data. *eLife*, *8*. https://doi.org/10.7554/eLife.49547

1. The direction of this association could be probed in future research by, for instance, experimentally manipulating the efficacy of instrumental actions through the reward probabilities, as done by Cartoni et al. (2015). [↑](#footnote-ref-1)
2. We used a range of 5 points because participants could not see the exact rating they provided of the rewards, i.e., they only saw the slider move on the visual analogue scale. Therefore, we considered using only differences scores of 0 to be overly strict. [↑](#footnote-ref-2)
